# Responders to low-dose ATG induce CD4^+^ T cell exhaustion in type 1 diabetes

**DOI:** 10.1172/jci.insight.161812

**Published:** 2023-08-22

**Authors:** Laura M. Jacobsen, Kirsten Diggins, Lori Blanchfield, James McNichols, Daniel J. Perry, Jason Brant, Xiaoru Dong, Rhonda Bacher, Vivian H. Gersuk, Desmond A. Schatz, Mark A. Atkinson, Clayton E. Mathews, Michael J. Haller, S. Alice Long, Peter S. Linsley, Todd M. Brusko

**Affiliations:** 1 Department of Pediatrics, College of Medicine, University of Florida, Gainesville, Florida, USA.; 2 Department of Pathology, Immunology, and Laboratory Medicine, University of Florida Diabetes Institute, Gainesville, Florida, USA.; 3 Benaroya Research Institute at Virginia Mason, Seattle, Washington, USA.; 4 Department of Biostatistics, University of Florida, Gainesville, Florida, USA.

**Keywords:** Endocrinology, Immunology, Autoimmune diseases, Diabetes, Immunotherapy

## Abstract

**BACKGROUND:**

Low-dose anti–thymocyte globulin (ATG) transiently preserves C-peptide and lowers HbA1c in individuals with recent-onset type 1 diabetes (T1D); however, the mechanisms of action and features of the response remain unclear. Here, we characterized the post hoc immunological outcomes of ATG administration and their potential use as biomarkers of metabolic response to therapy (i.e., improved preservation of endogenous insulin production).

**METHODS:**

We assessed gene and protein expression, targeted gene methylation, and cytokine concentrations in peripheral blood following treatment with ATG (*n* = 29), ATG plus granulocyte colony–stimulating factor (ATG/G-CSF, *n* = 28), or placebo (*n* = 31).

**RESULTS:**

Treatment with low-dose ATG preserved regulatory T cells (Tregs), as measured by stable methylation of *FOXP3* Treg-specific demethylation region (*TSDR*) and increased proportions of CD4^+^FOXP3^+^ Tregs (*P* < 0.001) identified by flow cytometry. While treatment effects were consistent across participants, not all maintained C-peptide. Responders exhibited a transient rise in IL-6, IP-10, and TNF-α (*P* < 0.05 for all) 2 weeks after treatment and a durable CD4^+^ exhaustion phenotype (increased PD-1^+^KLRG1^+^CD57^–^ on CD4^+^ T cells [*P* = 0.011] and PD1^+^CD4^+^ Temra MFI [*P* < 0.001] at 12 weeks, following ATG and ATG/G-CSF, respectively). ATG nonresponders displayed higher proportions of senescent T cells (at baseline and after treatment) and increased methylation of *EOMES* (i.e., less expression of this exhaustion marker).

**CONCLUSION:**

Altogether in these exploratory analyses, Th1 inflammation-associated serum and CD4^+^ exhaustion transcript and cellular phenotyping profiles may be useful for identifying signatures of clinical response to ATG in T1D.

**TRIAL REGISTRATION:**

ClinicalTrials.gov NCT02215200.

**FUNDING:**

The Leona M. and Harry B. Helmsley Charitable Trust (2019PG-T1D011), the NIH (R01 DK106191 Supplement, K08 DK128628), NIH TrialNet (U01 DK085461), and the NIH NIAID (P01 AI042288).

## Graphical abstract

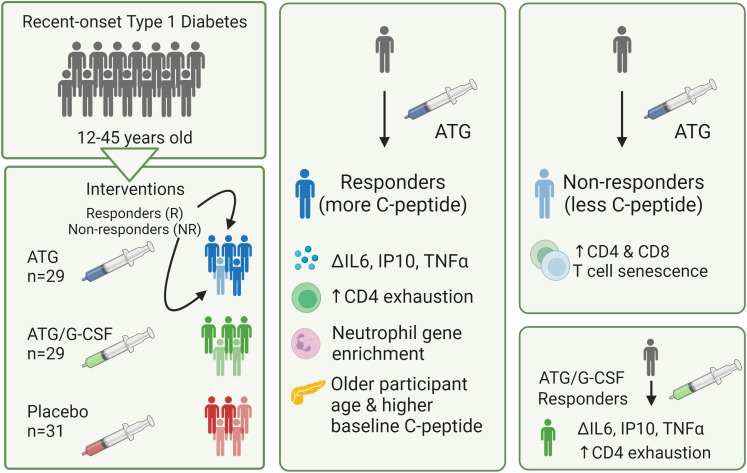

## Introduction

T cells are a key effector population contributing to the autoimmune pathogenesis of type 1 diabetes (T1D) (1, 2). A T cell–targeting immunomodulator, anti–thymocyte globulin (ATG), has been used in the settings of solid organ transplant, bone marrow transplant, and aplastic anemia for over 4 decades (at doses exceeding 10–20 mg/kg/course) and is associated with nearly complete immune suppression (3–5). In settings of therapeutic trials to impact T1D, ATG (Thymoglobulin, Sanofi Genzyme) was first used at a “high” dose of 6.5 mg/kg (START trial) but failed to demonstrate clinical efficacy in the primary endpoint, potentially due to the dose-associated depletion of both CD4^+^ T effector (Teff) and T regulatory (Treg) cell populations (6–8). In contrast, a single course of low-dose ATG (2.5 mg/kg) preserved C-peptide and reduced HbA1c in participants with recent-onset T1D for at least 2 years (9, 10).

Low-dose ATG avoided long-term immunosuppression and maintained beneficial regulatory subsets (e.g., Tregs) associated with immune tolerance (11, 12). Immune modulation with low-dose ATG augments the T cell compartment, leading to a reduction in CD4^+^ Teffs, increase in memory CD4^+^ T cells, and preservation of naive CD8^+^ T cells (9, 13). Nevertheless, due to the polyclonal nature of ATG, multiple facets associated with its beneficial mechanisms of action remain to be elucidated (14).

To better understand the role of low-dose ATG in T1D intervention efforts and to identify markers predictive of response, mechanistic exploratory analyses were performed on samples from the Type 1 Diabetes TrialNet (TN19) ATG/G-CSF New Onset clinical trial (ClinicalTrials.gov NCT02215200). Using genomic, epigenomic, transcriptomic, cytokine/chemokine, and immunophenotyping studies, we identified unique Th1 effector and T cell exhaustion immune signatures associated with clinical efficacy.

## Results

### Trial design, outcomes, and definition of therapeutic responders

The TrialNet low-dose ATG/G-CSF clinical trial in individuals with recent-onset T1D was a multisite, 3-arm, randomized, placebo-controlled, double-blind study that enrolled 89 pediatric and adult participants, 12–45 years old, as previously described (9) and reviewed in Supplemental Figure 1; supplemental material available online with this article; https://doi.org/10.1172/jci.insight.161812DS1 Eighty-eight participants underwent detailed mechanistic analysis.

The natural progression of T1D after diagnosis is accompanied by a decline in C-peptide over time as functional β cell mass is reduced (15). Notably, 1 year following treatment, 54% of ATG-treated participants had a C-peptide area under the curve (AUC) of no more than 15% below their baseline C-peptide AUC versus only 18% of the placebo- and 18% of the ATG/G-CSF–treated participants (*P* = 0.011 ATG vs. placebo; *P* = 1.000 ATG/G-CSF vs. placebo). In addition, 32% of participants in the ATG arm displayed an increase in the C-peptide AUC value at 1 year (i.e., produced more C-peptide than when they entered the trial). Thus, participants with a stimulated C-peptide at 1 year near their baseline value may be considered as having received therapeutic benefit.

**Figure 1:**
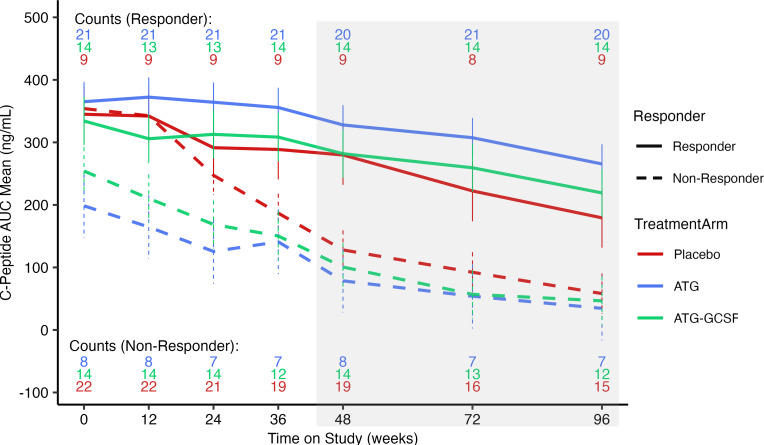
C-peptide AUC change over time for responders and nonresponders. The mean C-peptide AUC values for each treatment arm (ATG, blue; ATG/G-CSF, green; placebo, red) were disaggregated by responders (solid line) or nonresponders (dashed line). Response is based on the definition of an individual participant slope from baseline to 48 weeks being above the median (responders) or below the median (nonresponders) for the entire cohort, with the number of participants listed above or below the graph, respectively. The model included participant-level random effects terms for the intercept and slopes (16). The remaining clinical trial C-peptide AUC data from 48 weeks to 96 weeks are shaded in gray, as they were not part of the response definition. There were 582 samples collected, including 196 in the placebo group, 197 in the ATG group, and 189 in the ATG/G-CSF group. Among these, 279 samples were from the nonresponder group and 303 samples were from the responder group.

Here, responders and nonresponders were defined based on the change in C-peptide AUC during the 2-hour mixed meal tolerance test at baseline and 1 year (primary endpoint). The change in C-peptide AUC between these 2 time points, or slope, for each participant was determined (16). As no consistent definition of clinical response is used between T1D intervention trials (17), those above the median slope across all treatment arms combined (ATG, ATG/G-CSF, and placebo) were considered responders, and those below the median slope were considered nonresponders (Figure 1). By this definition, there were 21 responders in the ATG arm (*n* = 29), 14 in the ATG/G-CSF arm (*n* = 28), and 9 in the placebo arm (*n* = 31).

### Treatment effects

#### Transient lymphocyte reduction following low-dose ATG administration.

Consistent with past studies (10, 14), total lymphocyte counts from whole-blood complete blood count (CBC) decreased immediately in both treatment groups compared with placebo (*P* < 0.001 for both; Supplemental Figure 2). The CD4^+^ T cell count was lower in both treatment groups compared with placebo from week 4 through 48 (time points when samples were collected), and the CD8^+^ T cell count was unchanged (10). All participants in the ATG and ATG/G-CSF arms received methylprednisolone premedication (blinded) at the time of ATG infusion. Following glucocorticoid premedication, the absolute neutrophil count rose significantly on day 1 (*P* < 0.001) and remained significantly higher than the placebo arm at week 2 (*P* < 0.001; Supplemental Figure 2).

#### Differential neutrophil gene expression pattern distinguishes treated participants from placebo.

We sought to understand how ATG and ATG/G-CSF treatment affects whole-blood gene expression, as this approach has previously yielded novel insights into transcriptional networks (16, 18, 19). Therefore, we conducted gene set enrichment analysis (GSEA). There was increased expression of neutrophil-associated genes (azurocidin 1 [*AZU1*], neutrophil elastase [*ELANE*], proteinase 3 [*PRTN3*], cathepsin G [*CTSG*], chitotriosidase [*CHIT1*], matrix metallopeptidase 8 [*MMP8*], defensin A4 [*DEFA4*], and lactoferrin [*LTF*]). This was demonstrated by a normalized enrichment score (NES) log fold change (logFC) of 2.20 and 2.08 for ATG and ATG/G-CSF, respectively, compared with placebo (Benjamini-Hochberg–adjusted [BH-adjusted] *P* < 0.001 for both). This positive change was seen even when adjustment for the absolute neutrophil count (which can be affected by methylprednisolone administration; ref. 20) was included in the model.

We hypothesized that long-term differences in gene expression might result from epigenetic alterations in peripheral blood mononuclear cells (PBMCs). Therefore, we conducted pyrosequencing of targeted loci in immunoregulatory genes. We observed CpG demethylation of the myeloperoxidase gene (*MPO*; involved in neutrophil recruitment) (21) following ATG/G-CSF treatment at week 12 and week 48 across multiple CpG sites throughout the *MPO* gene (within exons 4, 8, and 10 as well as a 5′ upstream region and intron 3; *P* < 0.05 for all shown in Supplemental Figure 3). *MPO* gene expression (Supplemental Table 1) was also upregulated at week 2 in the ATG group (log_2_FC 3.20, BH-adjusted *P* < 0.001) and at weeks 2 and 12 in the ATG/G-CSF group (log_2_FC 2.30 and 1.18, BH-adjusted *P* < 0.001 and *P* = 0.002, respectively). Together, these data support the increased action of neutrophils as an effect of ATG treatment.

#### Th1 cytokine surge induced by ATG.

**Figure 2:**
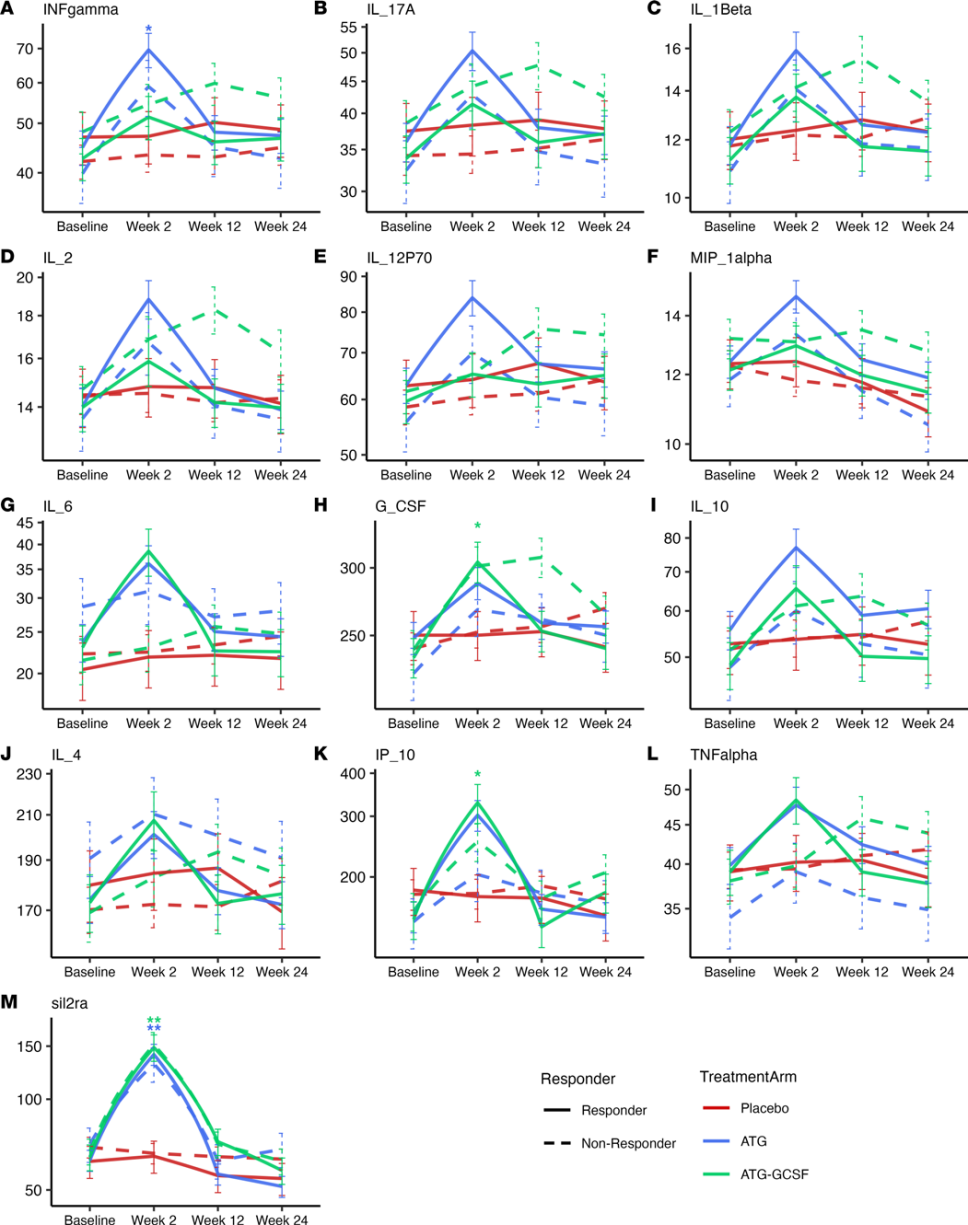
ATG and ATG/G-CSF transiently induce Th1 and innate cell cytokines. Cytokine log_2_ concentration (pg/mL) over time by treatment arm and responder/nonresponder status for (**A**) INF-γ, (**B**) IL-17A, (**C**) IL-1β, (**D**) IL-2, (**E**) IL-12p70, (**F**) MIP-1α, (**G**) IL-6, (**H**) G-CSF, (**I**) IL-10, (**J**) IL-4, (**K**) IP-10, (**L**) TNF-α, and (**M**) sIL-2RA. Blue lines represent the ATG arm, green lines the ATG/G-CSF arm, and red lines the placebo arm. Post hoc ANOVA (concentration log_2_ transformed) testing was done to determine differences in ATG versus placebo and ATG/G-CSF versus placebo. *P < 0.05, **P < 0.001. Solid lines denote responders and dashed lines nonresponders. Statistical comparisons between responders and nonresponders are not shown here. For panels **A**–**M**, there were 348 samples, comprising 87 samples at baseline, 87 samples at week 2, 87 samples at week 12, and 86 samples at week 24.

We next assessed whether circulating biomarkers denoted any innate or adaptive immune changes by conducting multiplexed cytokine/chemokine analysis of serum at baseline as well as 2, 12, and 24 weeks after treatment. The resulting data support a T cell–mediated treatment effect, with an increase in Th1, Th17, and innate inflammatory cytokines occurring at week 2 (Figure 2). ATG monotherapy induced IFN-γ and soluble IL-2 receptor α (sIL-2RA) — these concentrations were higher than placebo-treated individuals at 2 weeks (*P* < 0.05). Those who received ATG/G-CSF had higher cytokine concentrations of sIL-2RA (*P* < 0.001), G-CSF, and IP-10 (*P* < 0.05 for both) at 2 weeks compared with those who received placebo. While this cytokine surge can be a sign of Teff activation, lymphocyte counts (Supplemental Figure 2) and T cell–specific transcripts (Supplemental Table 1) demonstrate overall Teff depletion, suggesting that the reduced cell population is transiently activated.

**Figure 3:**
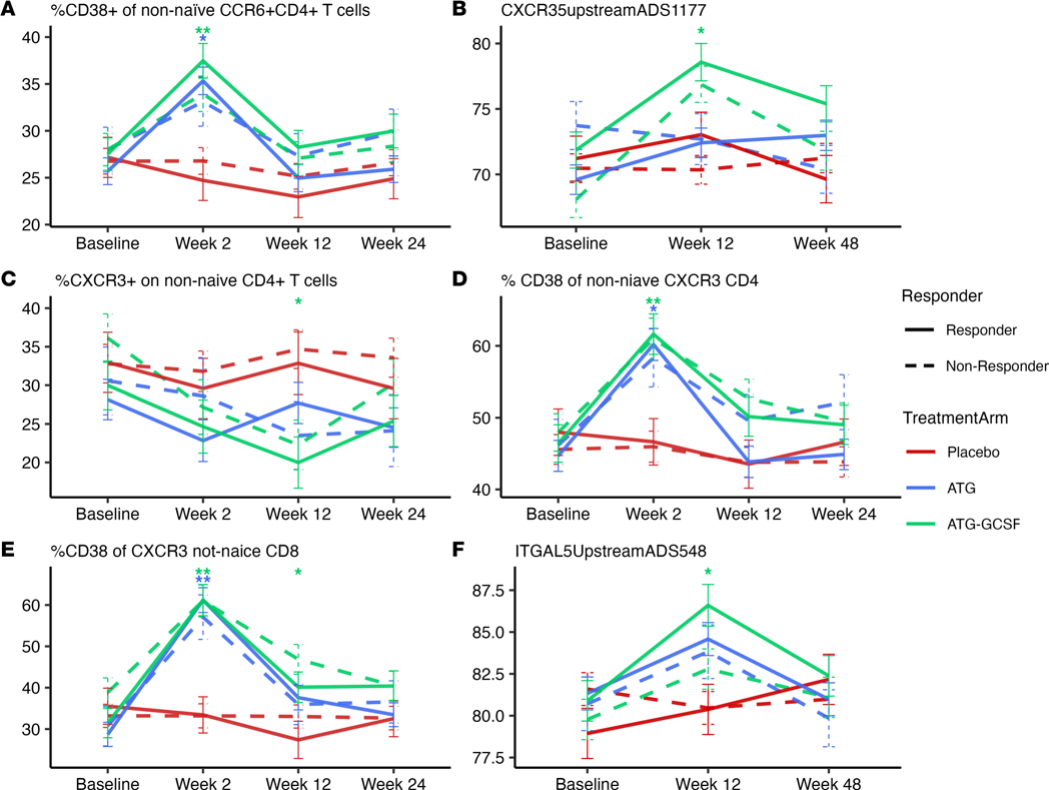
Th17 and Th1 augmentation following ATG and ATG/G-CSF denote this treatment effect is part of the mechanism of action in T1D. Blue lines represent the ATG arm, green lines the ATG/G-CSF arm, and red lines the placebo arm. Solid lines denote responders and dashed lines nonresponders. Panels depict (**A**) activated Th17 cells (percentage CD38^+^ of non-naive CCR6^+^CD4^+^ T cells) via flow cytometry (*n* = 322 samples over 4 time points), (**B**) median percentage methylation of the *CXCR3* 5′ upstream region (in the promoter) (*n* = 257 samples over 3 time points), (**C**) percentage of CXCR3^+^CD45RO^+^CD4^+^ T cells (*P* = 322), (**D**) percentage of activated CD4^+^ T cells (percentage CD38^+^ of non-naive CXCR3^+^CD4^+^ T cells) (*n* = 322), (**E**) percentage of activated CD8^+^ T cells (percentage CD38^+^ of non-naive CXCR3^+^CD8^+^ T cells) (*n* = 322), and (**F**) methylation of the *ITGAL* 5′ upstream region (*n* = 259). Several treatment effects were noted via post hoc ANCOVA (*P < 0.05, **P < 0.001). Statistical testing comparing responders and nonresponders is not demonstrated here.

In addition to Th1 activation signals, activated Th17 cells (percentage CD38^+^ of non-naive CCR6^+^CD4^+^ T cells via flow cytometry) also demonstrated a treatment effect following ATG (*P* = 0.001) and ATG/G-CSF (*P* < 0.001; Figure 3A) at 2 weeks compared with placebo. While a direct role for Th17 cells in autoimmune diabetes is debated in the NOD mouse model (22, 23), our data demonstrate that these cells are affected by ATG in T1D.

#### Teff trafficking and adhesion markers reduced following ATG and ATG/G-CSF.

We hypothesized that treatment with ATG may result in long-term epigenetic changes associated with persistent alterations in subset frequency and phenotype. We conducted targeted methylation as well as flow cytometric analysis covering a broad group of markers to corroborate the T cell treatment effects identified.

The change in median percentage methylation of regulatory gene regions (CpG islands) from baseline to 12 weeks and 48 weeks was assessed, with the greatest changes occurring over the first 12 weeks after treatment. Targeted bisulfite sequencing revealed increased methylation of the *CXCR3* promoter region (5′ upstream) in ATG/G-CSF–treated participants compared with placebo-treated participants across all time points together (*P* = 0.017), which is driven by changes at week 2 (*P* = 0.001; Figure 3B). Methylation of this region is likely associated with reduced gene expression, as is convention. Differences in methylation by sex (due to X chromosome inactivation) were accounted for in the model, as several genes were differentially methylated by sex, including that encoding the chemokine receptor CXCR3, which plays an important role in Teff trafficking and function (1, 24). Those treated with ATG/G-CSF had a lower percentage of CXCR3^+^ on non-naive CD4^+^ T cells (*P* = 0.005 at 12 weeks; Figure 3C). Of note, transient activation (measured via percentage CD38^+^) of CXCR3^+^CD4^+^ (ATG *P* = 0.001, ATG/G-CSF *P* < 0.001) and CD8^+^ (ATG and ATG/G-CSF *P* < 0.001 for both) T cells increased at 2 weeks (Figure 3, D and E). However, the overall abundance of CXCR3^+^CD4^+^ T cells was reduced (*P* ≤ 0.001 for both treatment arms; Supplemental Figure 4A), with CXCR3^+^CD8^+^ T cell counts similar between all 3 treatment groups (Supplemental Figure 4B).

In whole blood, *CXCR3* expression was unchanged between treated and placebo groups at both 2 and 12 weeks. However, the expression of *TBX21* (T-bet), an important transcription factor in Th1 lineage commitment (25), was decreased at 2 weeks following both ATG and ATG/G-CSF (Supplemental Table 1). CD11A (*ITGAL* [5′ upstream region]), which plays a role in CD4^+^ T cell adhesion and trafficking through T-bet stimulation (26, 27), demonstrated increased methylation over the placebo group at week 12 (ATG *P* = 0.065; ATG/G-CSF *P* = 0.013), with subsequent return to pretreatment levels by week 48. This is shown in Figure 3F. In contrast to these findings, *ITGAL* gene expression was overall unchanged except at 12 weeks following ATG where expression was higher than the placebo group (*P* = 0.048; Supplemental Table 1). Other cell adhesion and trafficking genes (*CD40LG* and *CCR4*) demonstrated reduced expression following both treatments (Supplemental Table 1). Thus, while T cell activation may be increased transiently as noted above, markers of T cell adhesion and trafficking are reduced.

#### Tregs remained unchanged in bulk PBMCs following ATG treatment.

**Figure 4:**
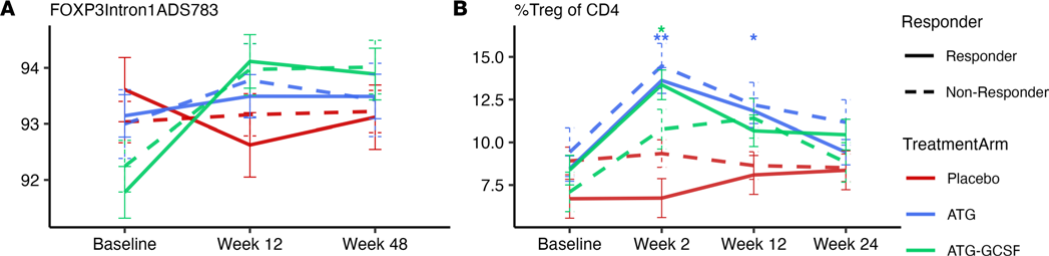
Differential effect of ATG and ATG/G-CSF on Tregs. Blue lines represent the ATG arm, green lines the ATG/G-CSF arm, and red lines the placebo arm; solid lines denote responders and dashed lines nonresponders. Panels depict (**A**) the median percentage methylation of the *TSDR* region of *FOXP3* (*n* = 255 samples over 3 time points) and (**B**) the percentage of FOXP3^+^CD4^+^ T cells via flow cytometry (*n* = 279 samples over 4 time points). Treatment effects were assessed via post hoc ANCOVA (*P < 0.05, **P < 0.001). In panel **A**, there was not a significant difference between ATG-treated participants and placebo- or ATG/G-CSF–treated and placebo at any single time point. However, there was a marked rise in the percentage methylation of the *FOXP3* *TSDR* from baseline to 12 weeks (and baseline to 48 weeks) in those in the ATG/G-CSF arm (*P* < 0.001 for both).

As noted above, changes in conventional CD4^+^ T cells were apparent following ATG treatment. Hence, we assessed the impact of treatment on Tregs, which can alter the effective ratio of Treg/Teff cells (10). In the ATG monotherapy arm, there was preservation (i.e., no change) in the methylation of the *FOXP3* conserved noncoding seqeuence-2 (CNS-2) region, also known as the Treg-specific demethylated region (*TSDR*) (Figure 4A). However, methylation studies of Tregs can be limited due to the small numbers of Treg within larger bulk cell populations (28). Nevertheless, the concept of Treg preservation following ATG is supported across modalities given (a) stable gene expression of *FOXP3* (log_2_FC –0.33, *P* = 0.331; Supplemental Table 1) and (b) increase in percentage FOXP3^+^ Tregs (of total CD4^+^ T cells) following ATG as well as ATG/G-CSF (Figure 4B).

Expression of other Treg-related genes in addition to *FOXP3*, specifically *IL2RA* and *GATA3*, was negatively affected. However, this was more notable following ATG/G-CSF than ATG alone (Supplemental Table 1). Additionally, there was a significant rise in methylation at the *FOXP3* *TSDR* locus from baseline to week 12 and week 48 (*P* < 0.001 for both) following ATG/G-CSF (Figure 4A), indicating a likely reduction in transcription. Helios (*IKZF2*) is the transcription factor of the lineage-stable thymic Treg subset (29); methylation at this locus was not significantly different between treatment arms (Supplemental Figure 5), though gene expression was reduced following ATG/G-CSF (log_2_FC –0.53, *P* = 0.01; Supplemental Table 1). Overall, Tregs were spared from low-dose ATG-induced depletion, but this effect was attenuated when G-CSF was added.

### Response effects

#### Baseline characteristics of therapeutic responders.

**Figure 5:**
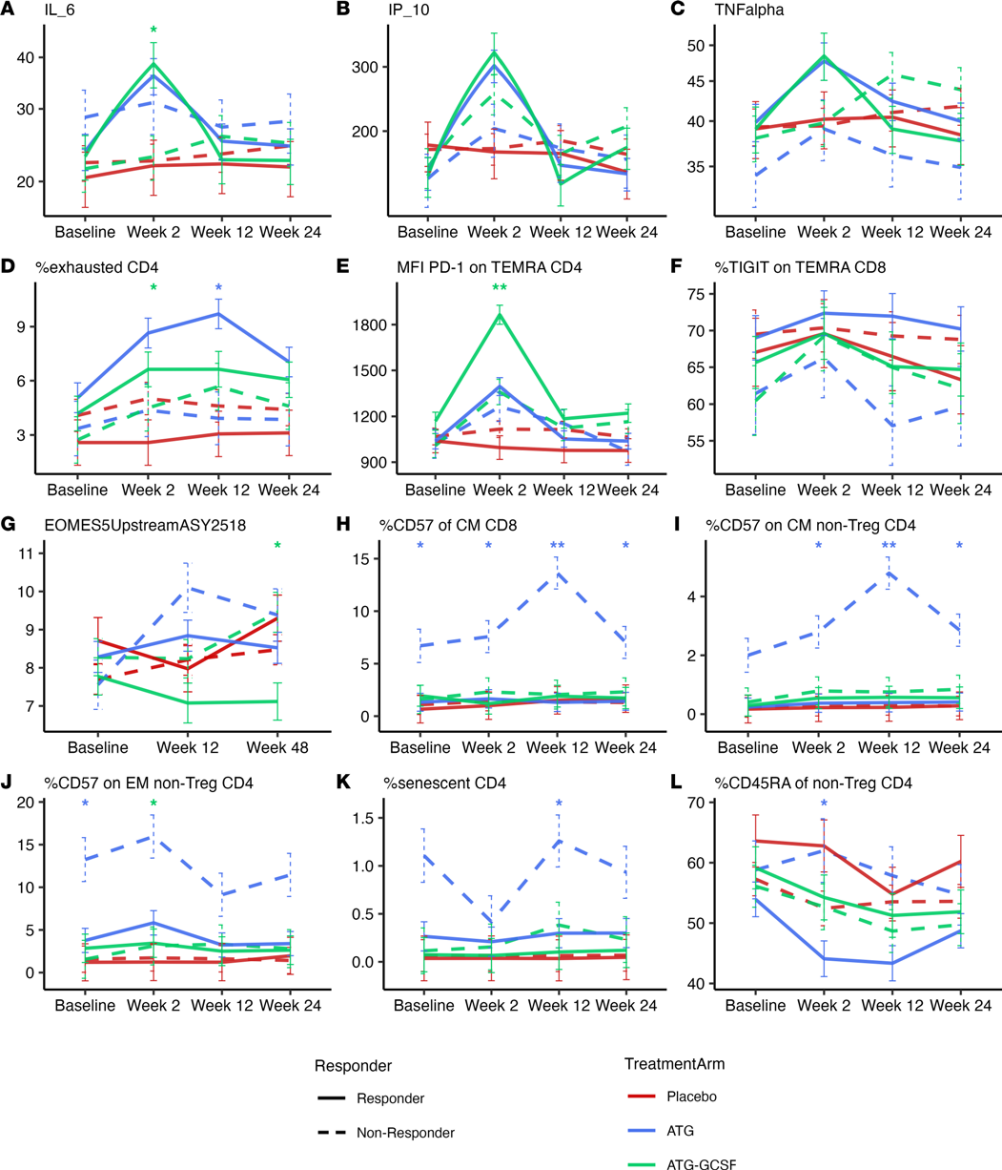
Responders identified via transient cytokine surge and exhaustion but not senescent T cell phenotype. Blue lines represent the ATG arm, green lines the ATG/G-CSF arm, and red lines the placebo arm. Solid lines denote responders and dashed lines nonresponders. The panels depict (**A**) IL-6 log_2_ concentration (*n* = 348 samples over 4 time points), (**B**) IP-10 log_2_ concentration (*n* = 348), (**C**) TNF-α log_2_ concentration (*n* = 348), (**D**) exhausted CD4^+^ T cells (percentage PD-1^+^KLRG1^+^CD57^–^ on CD4^+^ T cells) (*n* = 279), (**E**) median fluorescence intensity (MFI) of PD-1 on Temra CD4^+^ T cells (*n* = 264), (**F**) percentage TIGIT^+^ on Temra CD8^+^ T cells (*n* = 276), (**G**) median percentage methylation of *EOMES* 5′ upstream region (*n* = 252 samples over 3 time points), (**H**) percentage CD57^+^ of Tcm CD8^+^ T cells (*n* = 275), (**I**) percentage CD57^+^ on Tcm non-Treg CD4^+^ T cells (*n* = 276), (**J**) percentage CD57^+^ on Tem non-Treg CD4^+^ T cells (*n* = 277), (**K**) senescent CD4^+^ T cells (percentage CD57^+^KLRG1^+^ of PD-1^–^CD4^+^ T cells) (*n* = 279), and (**L**) naive CD4^+^ T cells (percentage CD45RA^+^ of non-Treg CD4^+^ T cells) (*n* = 322). *P < 0.05, **P < 0.001 by post hoc ANCOVA testing for comparisons between responders and nonresponders within a treatment arm. For panels **A** and **B**, there was a significant increase in cytokine concentration from baseline to 2 weeks and significant fall from 2 weeks to both 12 and 24 weeks in responders (*P* < 0.001 for all). In panel **C**, responders also demonstrated a significant rise from baseline to 2 weeks (*P* = 0.013) and significant fall from 2 weeks to 24 weeks (*P* = 0.004).

Baseline responder characteristics are reported in Table 1. Demographic and genetic background (sex, human leukocyte antigen [HLA] haplotype, and genetic risk score 1 [GRS1]) did not differentiate responders from nonresponders. Nor was autoantibody status at time of study enrollment or Epstein-Barr virus (EBV) and cytomegalovirus (CMV) infection serostatus a determining factor of response. There was a trend toward lower zinc transporter 8 autoantibody (ZnT8A) titers in ATG responders (*P* = 0.065). Older individuals (mean age 19.6 versus 15.0 years) were more likely to be responders to ATG (*P* = 0.043), but no clear age difference was seen with the ATG/G-CSF arm. While the C-peptide AUC values at baseline were similar across all treatment arms (*P* = 0.393), ATG responders had a higher baseline C-peptide AUC compared with ATG nonresponders (*P* = 0.002). A similar trend between ATG/G-CSF responders and nonresponders was seen, but without statistical significance (*P* = 0.064). Age and baseline C-peptide are interrelated, as older individuals are likely to have higher C-peptide at onset (30). Without a clear demographic feature of response, immune measures are of interest to explore. To account for age effects, all immune analyses were adjusted for age of the participant (Figure 5, A–L).

#### Baseline immune profiles of therapeutic responders.

ATG nonresponders had an increased percentage of senescent T cells (percentage CD57^+^ effector memory [Tem] CD4^+^ and percentage CD57^+^ central memory [Tcm] CD8^+^ T cells) at baseline that persisted throughout 24 weeks following ATG (Figure 5, H and J) and was driven by a subset of individuals. Prior to age adjustment, ATG/G-CSF responders displayed demethylation of *MPO* (intron 3) and ATG/G-CSF nonresponders demonstrated increased methylation of *TNFRSF18* (also known as glucocorticoid-induced TNFR-related protein; GITR) at intron 4. However, after age was incorporated into the model these ATG/G-CSF findings were no longer significant (Supplemental Figure 6). No other immune measures suggested response prior to treatment, and none of these features are complementary.

#### Posttreatment immune profiles of therapeutic responders.

Three cytokine/chemokines demonstrated significant changes between time points in ATG and ATG/G-CSF responders (Figure 5, A–C). IL-6 and IP-10 (CXCL10) concentrations significantly rose from baseline to week 2 and, likewise, significantly fell from week 2 to week 12 and week 2 to week 24 (*P* < 0.001 for all). Similarly, the mean concentration of TNF-α significantly rose from baseline to 2 weeks (*P* = 0.013) and fell from 2 to 24 weeks (*P* = 0.004) in all responders. In addition, the absolute IL-6 concentration was elevated in ATG/G-CSF responders and lower in nonresponders, whereas ATG responders and nonresponders had a similar elevation in IL-6 at 2 weeks (Figure 5A). This significant oscillation of these proinflammatory cytokine/chemokine levels was not observed in the serum of nonresponders.

We found an increased percentage of exhausted CD4^+^ T cells (percentage PD-1^+^KLRG1^+^CD57^–^ on CD4^+^ T cells, *P* = 0.011 at 12 weeks; Figure 5D) in ATG responders. MFI of PD-1^+^ on effector memory cells reexpressing CD45RA (Temra) CD4^+^ T cells was also markedly increased in ATG/G-CSF responders compared with ATG/G-CSF nonresponders (*P* < 0.001; Figure 5E). Increased inhibitory receptor expression was also found on CD8^+^ T cells in ATG responders as compared with ATG nonresponders at week 12, specifically demonstrating an increased percentage of TIGIT expression on Temra CD8^+^ T cells (*P* = 0.047); however, after age correction this CD8^+^ signature was no longer apparent (*P* = 0.207; Figure 5E). In further support of a T cell exhaustion phenotype, we also noted that ATG/G-CSF nonresponders had higher methylation of *EOMES* (5′ upstream region) at week 48 compared with ATG/G-CSF responders (*P* = 0.013; Figure 5G) and demonstrated a marked rise in *EOMES* methylation from baseline to week 48 in both ATG and ATG/G-CSF nonresponders (*P* = 0.011). This suggests that *EOMES* gene expression (an exhaustion marker; refs. 31, 32) is reduced in nonresponders and that a posttreatment measure of CD4^+^ T cell exhaustion may categorize responders.

In contrast to this exhausted phenotype in responders, a phenotype of terminal differentiation (senescence) of T cells was found in nonresponders. In ATG nonresponders, specifically, there was increased CD57 on Tcm CD8^+^, total CD4^+^, and Tem CD4^+^ T cells (Figure 5, H–J). CD4^+^ T cells with a cellular phenotype of senescence (PD-1^–^KLRG1^+^CD57^+^CD4^+^ T cells, *P* = 0.033 at week 12; Figure 5K) were also noted in ATG nonresponders. As there was increased T cell differentiation following treatment, we correspondingly found decreased naive CD4^+^ T cells in ATG responders at 2 weeks (*P* = 0.043; Figure 5L).

Innate immune cells, specifically neutrophils, also demonstrated a response effect. The neutrophil-associated gene set described above (*AZU1*, *ELANE*, *PRTN3*, *CTSG*, *CHIT1*, *MMP8*, *DEFA4*, *LTF*) that noted enrichment following both ATG and ATG/G-CSF treatments demonstrated gene enrichment in ATG responders (not ATG/G-CSF responders). The GSEA NES logFC was 1.89 for ATG responders compared with ATG nonresponders (genes upregulated in ATG responders, BH-adjusted *P* = 0.003) and –0.84 for ATG/G-CSF responders compared with ATG/G-CSF nonresponders (BH-adjusted *P* = 0.664).

Together, this responder analysis suggests a T cell activation and exhaustion signature in responders across multiple modalities.

#### Exploratory multimodal immunologic markers for response prediction.

Finally, given the broad treatment effect of ATG along with signatures identified above, we sought to determine whether machine learning could aid in development of a therapeutic response signature (i.e., C-peptide preservation). Using random forest multivariable modeling, we combined disparate variables to classify a clinical responder. All participants from the ATG and ATG/G-CSF arms were included. One hundred thirty-six features of immune function and demographic data (HLA, GRS, autoantibody type, EBV/CMV serostatus, CBC, cytokines, flow cytometry, and gene methylation) were included from the baseline time point (with 132 features included at week 2 and 134 features at week 12; Supplemental Figure 7A). We conducted 100 iterations of 5-fold cross-validation using the random forest model to select the variables. Subsequently, the selected variables were utilized to construct a random forest model for predicting the clinical responder. The receiver operator characteristic (ROC) AUC, estimated through 5-fold cross-validation, yielded a value of 0.78 for the 28 variables selected at baseline (Supplemental Figure 7B). At week 2, 21 variables were chosen to build the prediction model, resulting in an AUC of 0.73 (Supplemental Figure 7C). Similarly, at week 12, 15 variables were selected with an AUC of 0.79 (Supplemental Figure 7D). Combining data from all 3 time points improved the prediction accuracy, yielding an AUC of 0.82 (Supplemental Figure 7E). In support of the significant individual measures we found, the combined model identified more cytokines, T cell methylation markers, and inhibitory receptor cell phenotypes with good prediction accuracy.

## Discussion

Low-dose ATG is an effective therapy to delay C-peptide loss in recent-onset T1D, and the majority of participants respond favorably to low-dose ATG administration (9, 10). While adverse effects from low-dose ATG are mild compared with high-dose courses used for acute transplant rejection (3–5, 14), side effects remain, including cytokine release syndrome and serum sickness (9, 13). Identification of those individuals who are most likely to respond would reduce unnecessary risk. We identified a CD4^+^ and CD8^+^ senescent T cell phenotype at baseline, irrespective of participant age, in those unlikely to respond to low-dose ATG. More robust signatures were found after treatment, including cytokine markers of Th1 activation, CD4^+^ T cell exhaustion, and neutrophil gene upregulation in responders to ATG. These findings could allow for precise immunotherapeutic selection to delay complete C-peptide loss for those with newly diagnosed T1D. In addition, responder signatures could allow for early identification of who would not benefit from repeat or similar courses of therapy.

We observed both transient and persistent immune changes following ATG monotherapy and when ATG was used in combination with G-CSF (i.e., treatment effects). With ATG monotherapy, these include a reduction in lymphocytes, with CD4^+^ Teffs being the primary target that shifted toward a memory phenotype. CD8^+^ T cells remained relatively stable, as has been shown previously (9). The percentage of Tregs, a key cell type in immune tolerance, was increased with stable gene expression and methylation of *FOXP3* following ATG monotherapy. However, in the combination therapy, G-CSF appeared to impact Treg-associated genes in a negative way, through increased methylation at the *FOXP3 TSDR* locus and reduced *FOXP3*, *IL2RA*, and *GATA3* transcript expression, which may have dampened its clinical effectiveness. Likewise, the IL-6 concentration was elevated in ATG/G-CSF responders compared with nonresponders. This difference was not seen in the ATG arm alone. IL-6 has been described as an effector molecule of G-CSF treatment and also plays a role in differentiation of naive CD4^+^ and CD8^+^ T cells to effector phenotypes, specifically promoting Th17 cells at the expense of peripheral Tregs (33, 34). Overall, similar Th1- and proinflammatory-type cytokines were stimulated in both treatment arms, although the cytokine surge was prolonged following combination treatment (through 12 weeks after the start of therapy). Increased time of elevated cytokine concentrations, while lymphocyte counts are recovering, may lead to activation of new naive T cell populations. However, following ATG, the cytokine milieu had mostly returned to baseline after 2 weeks, while lymphocyte counts were at their nadir.

We found an appreciable effect on neutrophils, even though the majority of the signal was related to T cells, including increased activation markers. An increase in absolute neutrophil count was expected following treatment with ATG, as methylprednisolone was used as a premedication, and glucocorticoids result in neutrophil demargination (20). There was no methylprednisolone-only group to definitively explore these differences. However, this effect was transient, resolving by approximately 4 weeks. Increased neutrophil activation and degranulation gene transcripts and demethylated CpG sites were identified following ATG/G-CSF treatment. Increased neutrophil gene expression has been associated with slower C-peptide loss in individuals with recent-onset T1D across 6 clinical trials (16), which we also see using GSEA for 8 neutrophil-related genes even after adjusting for the total neutrophil count. Immune reconstitution following ATG with mobilization of immune cells from the bone marrow may be faster for innate versus adaptive cells (35). We posit that myeloid-focused studies, including low-density granulocytes and myeloid-derived suppressor cells, are still needed to provide support, or lack thereof, for myeloid-derived cells in the function of ATG in T1D (36).

In comparison with the transient increase in CXCR3, low-dose ATG was found to persistently induce expression of coinhibitory receptors (PD-1, KLRG1) on T cells and, thus, exhaustion-like phenotypes in CD4^+^ T cell populations. Increased coinhibitory receptors on CD8^+^ T cells was also found in responders; however, after adjusting for age this was no longer the case. Methylation of the *EOMES* 5′ upstream gene locus, likely leading to reduced gene expression, was found in nonresponders, and EOMES is also involved in T cell exhaustion and dysfunction. However, this is a finding in whole blood that needs further evaluation. Additionally, functional studies are required to confirm the exhausted T cell phenotype. A CD8^+^ T cell exhaustion phenotype has been associated not only with slower progression to complete loss of insulin secretion following diagnosis (31), but also with response to teplizumab (anti-CD3 monoclonal antibody) in new-onset T1D (37, 38) and at-risk individuals (39), as well as alefacept (lymphocyte function–associated antigen 3–IgG fusion protein) in new-onset individuals (40). Overall, a common shift toward T cell exhaustion following treatment with agents such as teplizumab, alefacept, or ATG now display some congruence in terms of a positive responder signature in the setting of autoimmunity, in opposition to the unfavorable state of T cell exhaustion in the context of chronic infection or cancer (41). Additionally, we can postulate that the strong activation signal resulting from low-dose ATG may work in the immune system to either delete autoreactive precursors (subject to ongoing investigations), exhaust T cells, and/or simultaneously preserve Tregs. Autoreactive T cell receptors are often low to moderate affinity, or they would be deleted by thymic negative selection (42). We speculate that low-dose ATG may target those cells that are accessible in the periphery and push them down toward exhaustion, thus shifting the balance of autoreactive cells in favor of regulation.

Responders to low-dose ATG also demonstrated reduced naive CD4^+^ T cells after treatment, whereas with abatacept, a CTLA-4/Fc fusion protein, memory T cell populations were reduced in responders (43). This supports the notion that response is tied, at least somewhat, to the mechanism of action of these drugs — abatacept prevents the costimulation of naive T cells to become memory cells, whereas ATG may transiently activate naive or memory T cells and then deplete them or push them toward exhaustion, reducing the naive T cell population. Similar to abatacept in the new-onset T1D trial, however, is the identification of neutrophil-specific features of response (43). Finally, the role of demographic and metabolic data in therapeutic response should not be overlooked. Older age may have played a beneficial role in the ATG arm, but not the ATG/G-CSF or placebo arms. Participants with a higher starting C-peptide in the ATG arm were most likely to respond and this was a trend in the ATG/G-CSF and placebo arms as well. However, we cannot know whether this is merely an effect of age, as older individuals have higher C-peptide (30). This may or may not be due to personalized dosing differences. Future studies will explore the role ATG binding, clearance, or body mass index may play. Additionally, in Europe, the Innovative Approach Towards Understanding and Arresting Type 1 Diabetes (INNODIA) collaborative is enrolling the Minimum Effective Low Dose: Anti-human Thymocyte Globulin (MELD-ATG) study (ClinicalTrials.gov NCT04509791).

Response to ATG was not found to associate with the degree of serum sickness (9) or lymphocyte depletion. The immune features of responders we uncovered are unlikely to be a result of aging, as analyses were adjusted for age. Older participants (22–35 years old) in the high-dose ATG START trial (6, 44) also demonstrated C-peptide preservation, whereas the trial population as a whole did not. Other therapeutics — both B cell–directed (rituximab) and T cell–directed (teplizumab and abatacept) therapies — have demonstrated greater metabolic benefit in younger individuals (37, 43, 45, 46). Thus, there are inherent differences in how these therapies work in individuals with T1D that will allow for targeted treatment decisions.

There were caveats to our experiments that require further elucidation. The broad, exploratory nature of this analysis resulted in limited depth; however, single-cell analyses are underway. Also, it remains to be fully understood how drugs like ATG impact tissue-resident memory populations — constituting an area in need of additional study, through the development of novel in vivo imaging methods, to potentially increase long-term efficacy. Another limitation is the use of an arbitrary responder classification, as no consensus definition has been reached in the field, though this definition has been reported previously (16). Increased risk of type 1 error is possible, as corrections for multiple comparisons were only performed within an assay modality (e.g., methylation data), and not between assay modalities (e.g., methylation and cytokine data). When we utilized random forest modeling that can identify predictive features with low risk of overfitting (47), the performance was encouraging and overall results mirrored single-modality analyses. Small changes in percentage methylation of CpG sites were present, and without functional studies, clinical relevance determinations are limited. The broad nature of assays chosen did achieve our aim of identification of potential predictive markers for further study. However, bulk analysis of PMBCs can limit identification of changes in small cell populations, and single-cell assays are an important next step. Finally, cell surface markers were used to classify cell populations, but functional testing is needed to confirm activation and exhaustion states.

Targeted reduction of Teff infiltrating lymphocytes in addition to the transient (i.e., not prolonged or persistent) stimulation of Th1 and proinflammatory serum cytokine concentrations supports the beneficial effect (Table 2) of low-dose ATG, and to a lesser extent, ATG/G-CSF, in T1D (1, 48, 49). One immune marker that was identified to predict nonresponse to ATG was an increase in senescent cells (CD57^+^CD4^+^ Tem and CD57^+^CD8^+^ Tcm cells) at baseline. This same cell population was also seen after treatment and suggests a benefit in T cell exhaustion but not T cell senescence. Exhausted and senescent T cell populations as well as neutrophil signatures could be future biomarkers of ATG response following validation in another population. These exploratory analyses suggest that ATG may be optimized based on the immune profile and dose used.

## Methods

### Clinical trial.

A randomized, double-blind, placebo-controlled trial in recently diagnosed participants with T1D was conducted through the Type 1 Diabetes TrialNet clinical trial consortium as a multisite trial in the United States. Participants received either low-dose ATG, low-dose ATG plus G-CSF, or placebo in a 1:1:1 randomization as previously published (9). In summary, enrollment consisted of 89 participants, with 87 reaching the primary endpoint (the 2 participants who withdrew were in the ATG/G-CSF arm). Mechanistic analyses were conducted on all participants at baseline and the majority of participants across other time points (week 2, 12, 24, and 48) using assays (detailed below) to determine the immunological effects of each therapy, identify responder characteristics, and predict responders versus nonresponders.

### gDNA isolation.

Genomic DNA (gDNA) was isolated from whole blood using a QiaCube with QIAamp DNA Blood Mini Kit (Qiagen) according to the manufacturer’s instructions for high-throughput nucleic acid purification.

### Genetic analysis.

A custom Axiom array, including 975,000 genetic markers, was used to genotype gDNA from all participants for HLA and non-HLA T1D risk alleles (50, 51). HLA-DR-DQ diplotypes were imputed and GRS1 calculated per a previous protocol (52).

### EBV and CMV infectious history.

Participants were not randomized in the trial if active EBV and/or CMV infection was present (viral PCR) (9). Serological status at baseline and 1 year was assessed using EBV viral capsid antigen (VCA) IgG, EBV VCA IgM, EBV nuclear antigen (EBNA) IgG, CMV IgG, and CMV IgM via chemiluminescent immunoassays performed at University of Colorado (DiaSorin LIAISON). Past infection (including recent infection or reactivation) with EBV was characterized by (a) IgG^+^, IgM^–^, EBNA IgG^+^; (b) IgG^+^, IgM^+^, EBNA IgG^+^; or (c) IgG^–^, IgM^–^, EBNA IgG^+^. Prior CMV infection was characterized by IgG^+^ and IgM^–^ status (53).

### Cytokine/chemokine studies.

Cytokine and chemokine concentrations were measured in serum using a magnetic bead panel (Milliplex) on a Luminex MAGPIX according to the manufacturers’ instructions. The analytes measured (and minimum detectable concentrations) were G-CSF (1.8 pg/mL), IFN-γ (0.8), IL-10 (1.1), IL-12p70 (0.6), IL-17A/CTLA8 (0.7), IL-1β (0.8), IL-2 (1.0), IL-4 (4.5), IL-6 (0.9), IP-10/CXCL10 (8.6), MIP1α/CCL3 (2.9), and TNF-α (0.7). ELISA (BD Biosciences) was performed for sIL-2RA (sCD-25) in serum according to the manufacturer’s instructions. Modeling of data from time points at 0, 2, 12, and 24 weeks of the trial was conducted incorporating age, sex, time point, and treatment arm. Concentrations (pg/mL) were plotted over time by treatment arm and responder status (definition below).

### CpG methylation studies.

Epigenetic analysis of CpG methylation (EpigenDx) was conducted from gDNA with targeted Next-Gen bisulfite sequencing for the following genes of interest: *PDL2*, *IL2RA*, *IL2*, *IL10*, *CD274*, *MYC*, *CDKN1C*, *MAPRE2*, *RUNX1*, *TNFRSF25*, *TOLLIP*, *ITGAL*, *EST1*, *IKZF2*, *FOXP3*, *TNFRSF18*, *TNF*, *CTLA4*, *IRF8*, *S100A6*, *PDCD1*, *TIGIT*, *CTLA4*, *CXCR3*, *ARG1*, *MPO*, *TBX21*, *CD27*, and *EOMES*. Two hundred eighty-three CpG sites were assessed across 32 genes, with 231 sites retained (52 sites removed as >20% of data missing at the site). For visualizations, missing values were imputed per site to each site’s overall median. Thirteen CpG sites from 7 genes were excluded due to less than 50% of individuals having greater than 100 reads. *CD3D*/*CD3G* and *IL4* were analyzed via pyrosequencing (EpigenDx). Time points included 0, 12, and 48 weeks. Samples were removed that failed quality control (QC) for signal strength (relative light units), which included *ARG1*, *IL2*, *IL10*, and *IL4*. No batch effects were found during QC. Sex chromosome differences (variable methylation of inactivated X chromosome in females) were adjusted for in all modeling. Two genes analyzed reside on the X chromosome — *CXCR3* and *FOXP3* — while another gene, *TIGIT*, also demonstrated significant variable methylation by sex even though it is on chromosome 3. Individual CpG sites were analyzed in addition to gene locations of interest (e.g., promoters) within the gene. When methylation results from different CpG clusters within the same gene were disparate, we characterized each individual CpG and CpG cluster for likely dominance based on location within the gene such as *CXCR3* promoter versus gene-body, *TBX21* exon 3 (CCCTC-binding factor [CTCF]) versus intron 1, and *FOXP3* *TSDR* versus the rest of intron 1. Normalization of data across percentage CD3 methylation did not alter interpretations, and thus is not shown.

### Flow cytometry.

CBC including CD4^+^ and CD8^+^ cell counts were measured in whole blood using an FC500 with 4-color fluorescent murine monoclonal antibody reagents (Beckman Coulter). Flow cytometry panels for T cells, myeloid cells, and NK cells were predetermined (Supplemental Table 2) and published previously (9, 54). In summary, cryopreserved PBMCs were thawed and then incubated with viability dye (LIVE/DEAD blue fixable stain kit, Thermo Fisher Scientific) and Human TruStain FcX blocking solution (BioLegend) prior to staining and acquisition on an LSRFortessa (BD Biosciences). Previously used QC measures (16) included 8-peak Rainbow Calibration beads (Spherotech) to adjust PMT voltages, and use of a technical control from 1 individual run concomitantly on each acquisition day. All longitudinal samples from a given individual were acquired on the same day. Criteria for analysis exclusion were fewer than 25 events for frequency and fewer than 50 events for MFI reporting. Data were analyzed and hierarchically gated using FlowJo software version 9.9.6 (Tree Star). Redundant populations between panels, negative gates, and samples with high technical variability (CV > 40% in internal control) were culled from the data set. Two hundred eight cell populations were studied over the first 6 months of the trial, specifically at baseline, 2 weeks, and 24 weeks.

### Transcriptomic analysis.

Bulk RNA-seq from whole blood and analysis of differential gene expression by modules (neutrophil activation modules, CXCR1.mod, and CD3E.mod for neutrophil-specific and T cell–specific genes) was completed (55). Time points included were 0, 2, 24, and 48 weeks. Whole blood was collected in Tempus Blood RNA Tubes (Thermo Fisher Scientific), RNA was extracted using MagMax for Stabilized Blood Tubes RNA Isolation Kit (Thermo Fisher Scientific), followed by globin reduction using GlobinClear Human (Thermo Fisher Scientific). To generate sequencing libraries, total RNA (0.5 ng) was added to reaction buffer from the SMART-Seq v4 Ultra Low Input RNA Kit for Sequencing (Takara), and reverse transcription was performed followed by PCR amplification to generate full-length amplified cDNA. Sequencing libraries were constructed using the NexteraXT DNA sample preparation kit (Illumina) to generate Illumina-compatible barcoded libraries. Libraries were pooled and quantified using a Qubit Fluorometer (Life Technologies). Dual-index, single-read sequencing of pooled libraries was carried out on a HiSeq 2500 sequencer (Illumina) with 58-base reads, using HiSeq v4 Cluster and SBS kits (Illumina) with a target depth of 5 million reads per sample. Base calls were processed to FASTQs on BaseSpace (Illumina), and a base call quality-trimming step was applied to remove low-confidence base calls from the ends of reads.

### Responder analysis and multivariable modeling.

Clinical responders and nonresponders were defined as those with slope of C-peptide AUC above (responders) or below (nonresponders) the median for the study cohort where models included individual-level random effects terms for the intercept and slopes (16). This slope was determined for each participant as the change in C-peptide AUC from baseline to 1 year.

Multivariable modeling using random forests was done to assimilate all markers and identify a combination of variables that predict response and determine their accuracy at baseline, week 2, week 12, or all 3 time points. Variables included in the multivariable model were (Supplemental Figure 7A) age, sex, treatment arm (ATG or ATG/G-CSF), autoantibodies at baseline, EBV and CMV serostatus at baseline, HLA (DR3/4, DR3, DR4), GRS1, absolute lymphocyte count, absolute neutrophil count, absolute monocyte count, cytokine concentrations (12 cytokines), percentage methylation (28 genes), and 81 cell subsets defined by flow analytes defined in Supplemental Table 2. As the GRS1 includes imputed HLA as part of the risk assessment, these variables are highly related and if either had been significant in this exploratory analysis it would need further assessment to determine validity.

We initiated the variable selection process using a random forest model with 5-fold cross-validation. Out of the 136 variables at baseline, on average, 32 were found to be significant in characterizing responders based on their variable importance scores. To ensure the stability of our selection, we repeated the 5-fold cross-validation random forest procedure 100 times, resulting in 28 variables with an inclusion probability above 50%. For the baseline variables, the median ROC AUC was 0.60. Moving to week 2, the model selected an average of 30 variables, with 21 demonstrating an inclusion probability above 50% and a median ROC AUC of 0.59. Similarly, at week 12, 21 variables were selected, and 15 were chosen with a median ROC AUC of 0.58. When combined from all 3 time points, the model selected an average of 50 variables, with 30 demonstrating an inclusion probability above 50% and a median ROC AUC of 0.59. These results indicate that the selected variables in these models generally exhibited predictive capability for distinguishing between responders and nonresponders.

Subsequently, we constructed new random forest models using the variables that had an inclusion probability above 50% to predict the clinical responder and reported those prediction accuracy ROC AUC values in the Results above. Five-fold cross-validation was performed 100 times and those variables with greater than 50% inclusion probability were displayed using the VSURF v1.1.0 R package (https://cran.r-project.org/web/). The ROC AUC was calculated using the pROC v1.17.0.1 R package. Flow cytometry variables with minimal variation across participants were removed. Methylation variables with more than 20% missing values were removed. Missing values were replaced with the median. Within the cytokine, flow cytometry, and methylation data, highly correlated values (Spearman’s correlation >0.9) were used as a single variable (dimension reduction using principal component 1).

### Statistics.

Proportional comparison of C-peptide production between treated and placebo groups was conducted via Fisher’s exact test. Four participants had incomplete serum for cytokine/chemokine analysis (missing at least one of baseline, week 2, week 12, or week 24). Cytokine levels were natural log transformed. Modeling for the cytokine analysis, CBC analysis, flow cytometry analytes, and methylation studies utilized mixed-model ANOVA, treating participants as a random effect and all other variables as fixed effects controlling for the effects of age and sex. Models were fit using the lme4 v1.1-27 R package. When type II ANCOVAs were significant at the 0.05 level, post hoc group 2-tailed *t* tests were conducted within each variable to identify group differences significant at the 0.05 level. Adjustment for multiple testing within each significant independent variable was done using Tukey’s method and the adjustment within each significant interaction variable was corrected by Šidák’s method. In addition, the comparison between responders versus nonresponders within a treatment arm via 2-way mixed ANCOVA was corrected by Šidák’s method.

Differential expression analysis used DESeq2 v1.34.0 R package. The genes with total expression counts less than 5 were filtered out from the bulk RNA data and then the RNA data were normalized by the generalized linear model. Age and sex were controlled during differential expression analysis. Neutrophil-related genes were also controlled using the absolute neutrophil count. Wald’s test was applied to identify the differentially expressed genes and BH (56) was utilized to adjust *P* values. The fgsea v1.20.0 R package was used to test whether the top-ranked differentially expressed genes had overrepresented specific gene sets. *P* values were corrected by BH. Significance for *P* values was set at less than 0.05; however, as this was an exploratory analysis, correction for multiple testing was not conducted across modalities.

For line charts, the marginal means were extracted using the emmeans v1.6.0 R package and plotted using the ggplot2 v3.3.3 R package. Analyses were conducted using R v4.0.4.

### Study approval.

Human samples were analyzed in a deidentified fashion. The clinical trial from which these samples were obtained was approved by each site’s Institutional Review Board and all participants (or their legal representative in the case of minors) provided written informed consent before participating.

### Data availability.

Data from the Type 1 Diabetes TrialNet (TN19) ATG/G-CSF New Onset clinical trial (NCT02215200) reported here are available for request at the NIDDK Central Repository (NIDDK-CR) website, Resources for Research (R4R), https://repository.niddk.nih.gov/ Supporting data values for each figure were made available in the Supporting Data Values file.

## Author contributions

TMB, PSL, SAL, and MJH conceptualized this study. LMJ, KD, and LB analyzed the data and wrote the manuscript, with primary drafting by LMJ (listed first, followed by KD and LB, who provided similar and essential input). JM (sample preparation), DJP (HLA and GRS1 determination), VHG (transcript data), JB (methylation data) generated and analyzed the data, and contributed to discussion. XD and RB performed statistical analysis. DAS, MAA, and CEM reviewed the data and contributed to discussion and the manuscript.

## Supplementary Material

Supplemental data

Supporting data values

## Figures and Tables

**Table 1:**
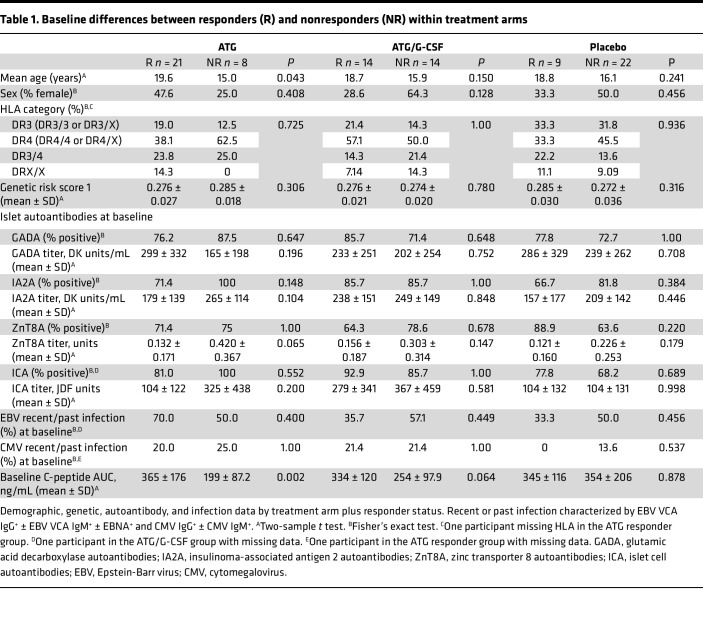
Baseline differences between responders (R) and nonresponders (NR) within treatment arms

**Table 2:**
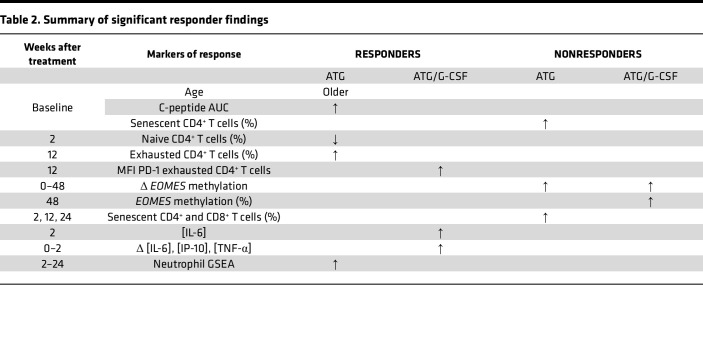
Summary of significant responder findings

## Version 1 07/11/2023

In-Press Preview

## Version 2 08/22/2023

Electronic publication
