# Supplementary material for: Responders to low-dose ATG induce CD4+ T cell exhaustion in type 1 diabetes
Source: JCI Insight. 2023 Aug 22;8(16):e161812. doi: 10.1172/jci.insight.161812 (PMC10543726; doi:10.1172/jci.insight.161812)
Supplement: Supplemental data [file jciinsight-8-161812-s061.pdf]

Supplemental Data

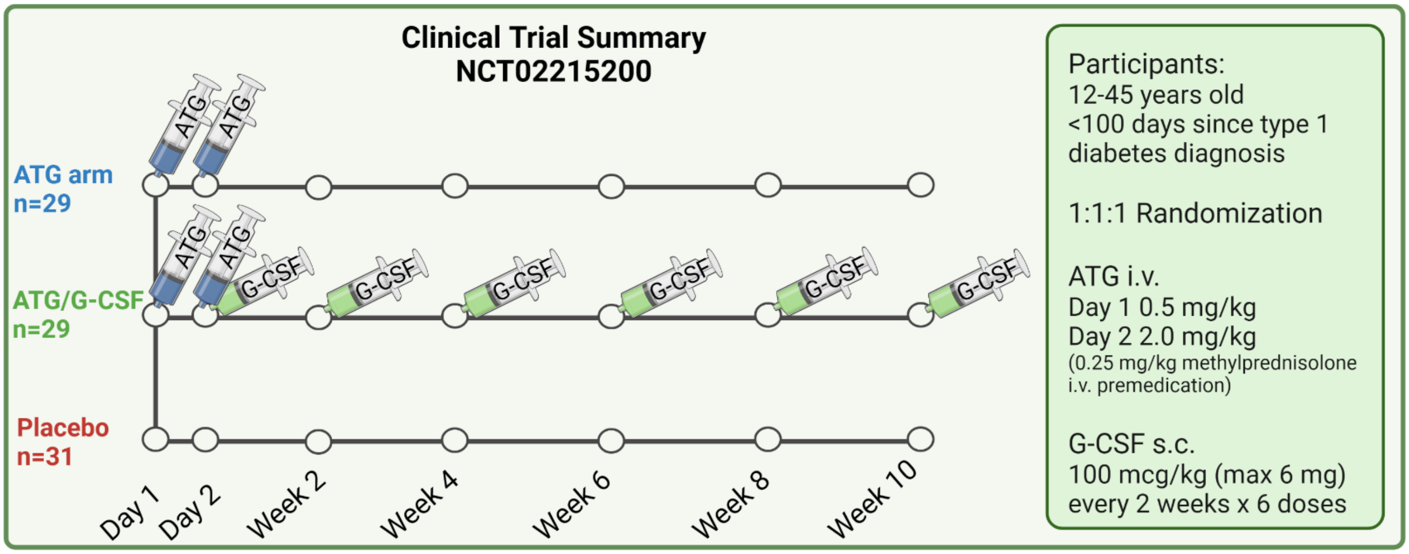

Supplemental Figure 1: Diagrammatic representation of drug administration by treatment arm. ATG, anti-thymocyte globulin; G-CSF, granulocyte-colony stimulating factor; i.v., intravenous; s.c., subcutaneous.

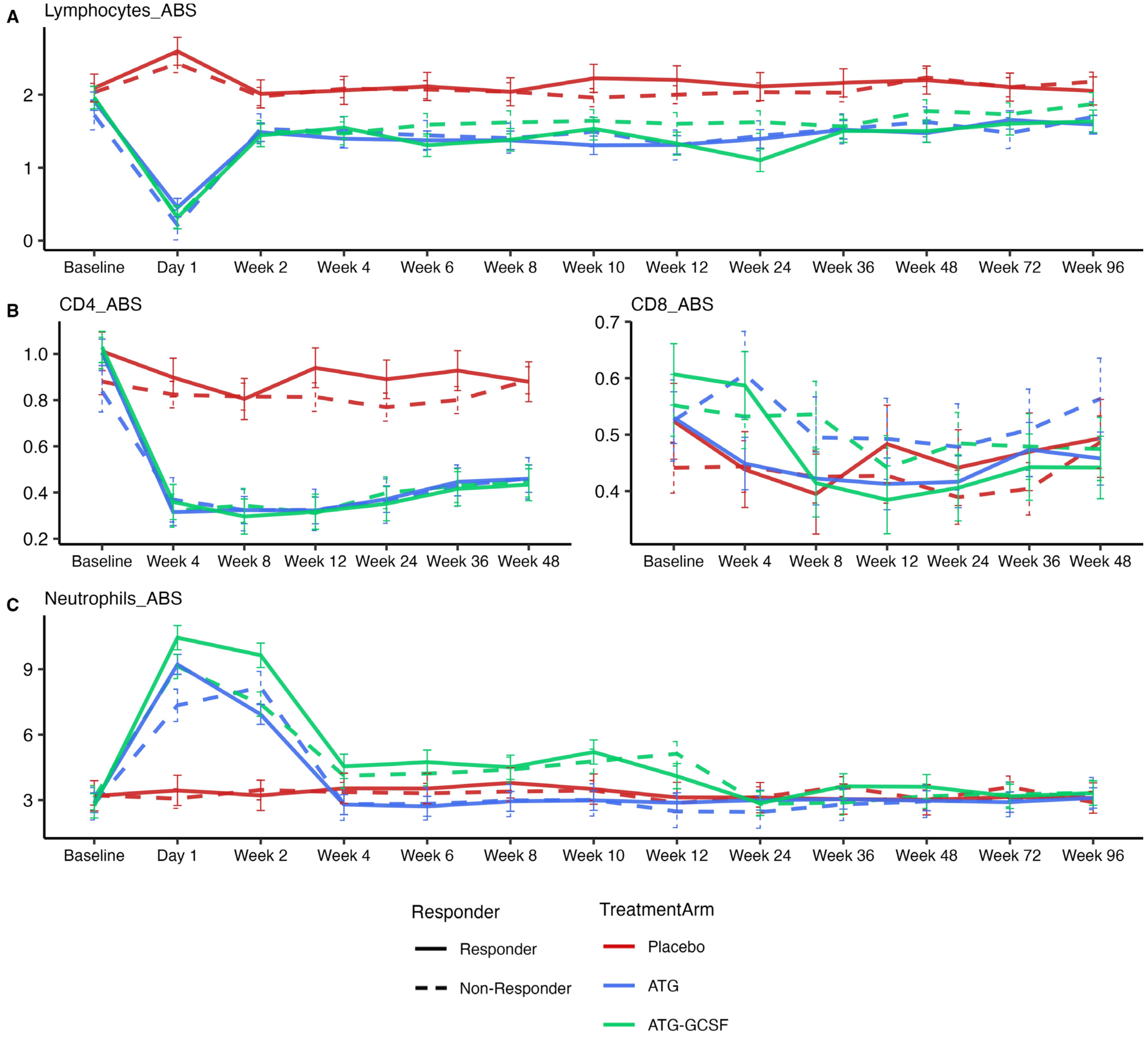

<u>Supplemental Figure 2: Lymphocyte counts, lymphocyte subsets, and neutrophil counts by treatment arm and responder/non-responder status.</u> Plot by treatment arm and responder status of the (A) absolute lymphocyte count (10^3 cells/µL), (B) absolute CD4 count (10^3 cells/µL) and absolute CD8 count (10^3 cells/µL), and (C) absolute neutrophil count (10^3 cells/µL). Blue lines represent the ATG arm, green lines the ATG/G-CSF arm, red lines the Placebo arm. Post-hoc-ANCOVA comparing responders (solid lines) versus non-responders (dashed lines) within each treatment arm was not significant. N=3 subjects with missing CBC values (1 from Placebo Responders at 2 weeks; 1 from ATG Responders at 72 weeks; 1 from ATG Non-responders at 72 weeks). ABS, absolute.

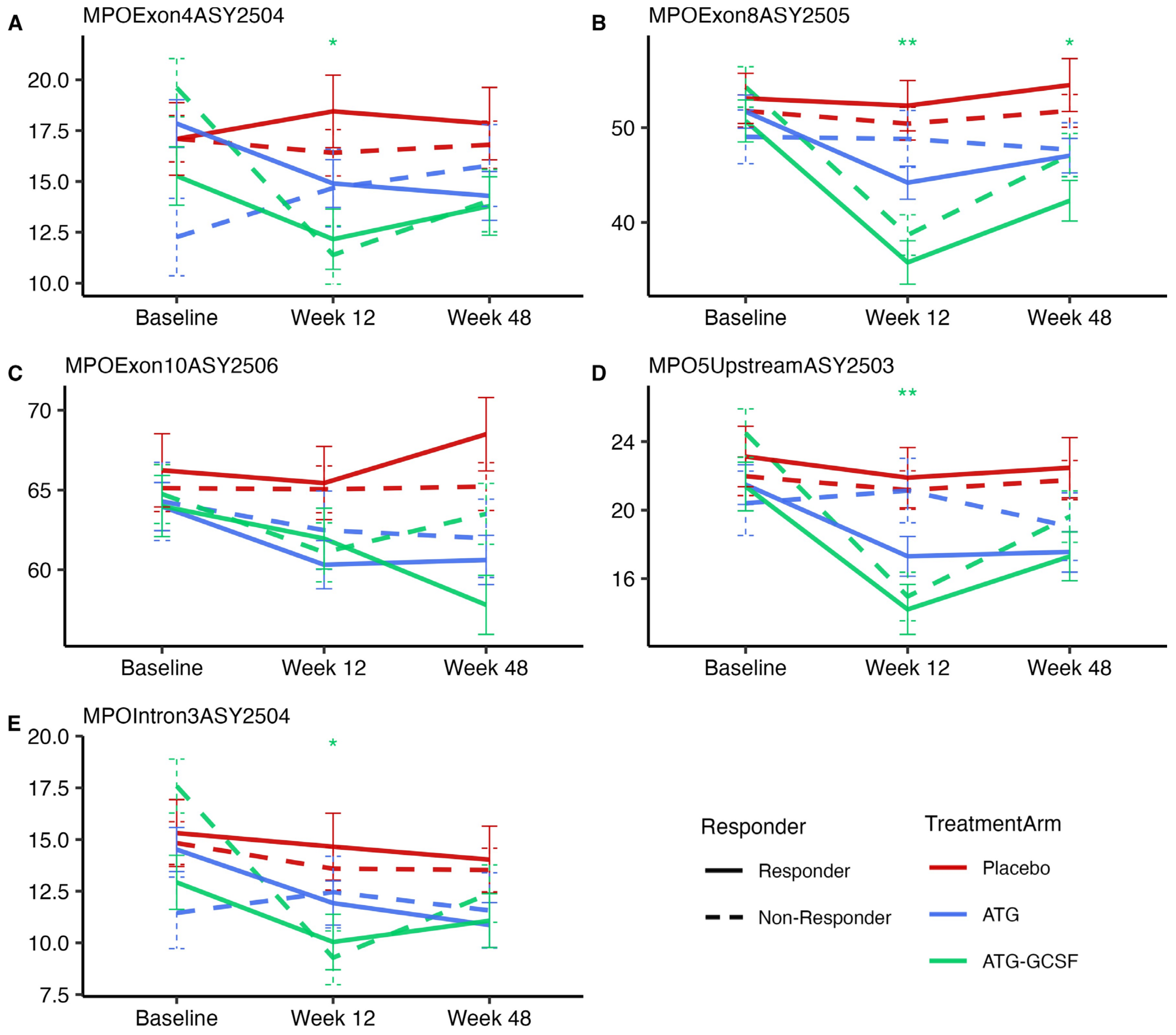

Supplemental Figure 3: Myeloperoxidase (MPO) methylation changes across multiple gene regions (CpG islands). Blue lines represent the ATG arm, green lines the ATG/G-CSF arm, red lines the Placebo arm. Solid lines denote responders and dashed lines non-responders. Comparison between treatment arms (not responders) was conducted using ANCOVA analyses followed by post-hoc group t-tests, p<0.05*, p<0.001**.

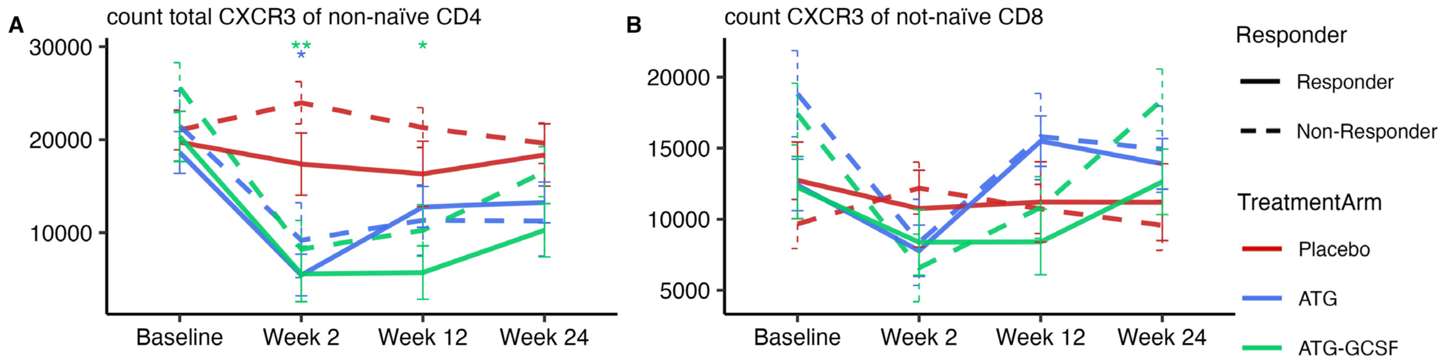

<u>Supplemental Figure 4: $CXCR3^{+}CD4^{+}$, but not $CXCR3^{+}CD8^{+}$, T cell numbers decrease following ATG and ATG/G-CSF.</u> Blue lines represent the ATG arm, green lines the ATG/G-CSF arm, red lines the Placebo arm. Solid lines denote responders and dashed lines non-responders. Comparison between treatment arms (not responders) was conducted using ANCOVA analyses followed by post-hoc group t-tests, $p<0.05$*, $p<0.001$**.

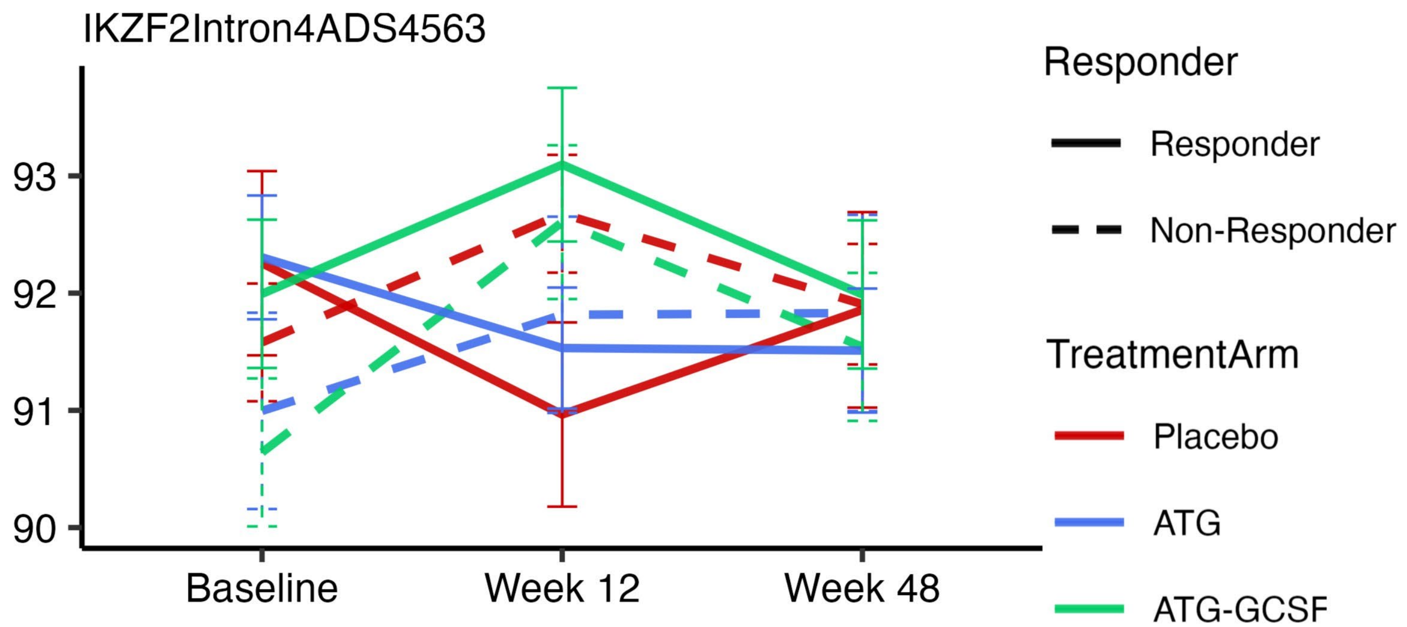

<u>Supplemental Figure 5: Helios (IKZF2) methylation changes not significant.</u> Blue lines represent the ATG arm, green lines the ATG/G-CSF arm, red lines the Placebo arm. Solid lines denote responders and dashed lines non-responders.

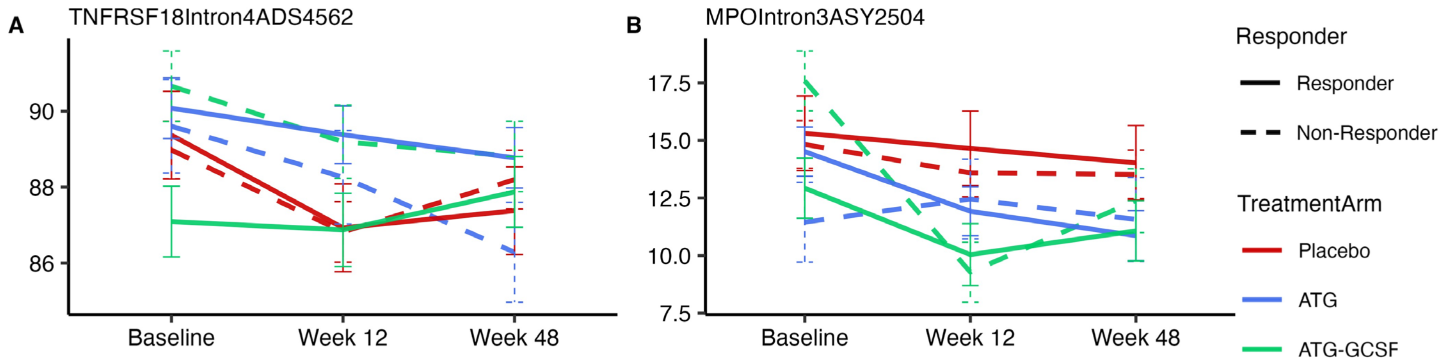

<u>Supplemental Figure 6: Additional methylation trends in responders.</u> Blue lines represent the ATG arm, green lines the ATG/G-CSF arm, red lines the Placebo arm. Solid lines denote responders and dashed lines non-responders. (A) median percent methylation of the *TNFRSF18* (*GITR*) at intron 4 ($p=0.066$ at baseline for ATG/G-CSF responders versus non-responders). and (B) methylation of *MPO* intron 3 ($p=0.114$ at baseline for ATG/G-CSF responders versus non-responders).

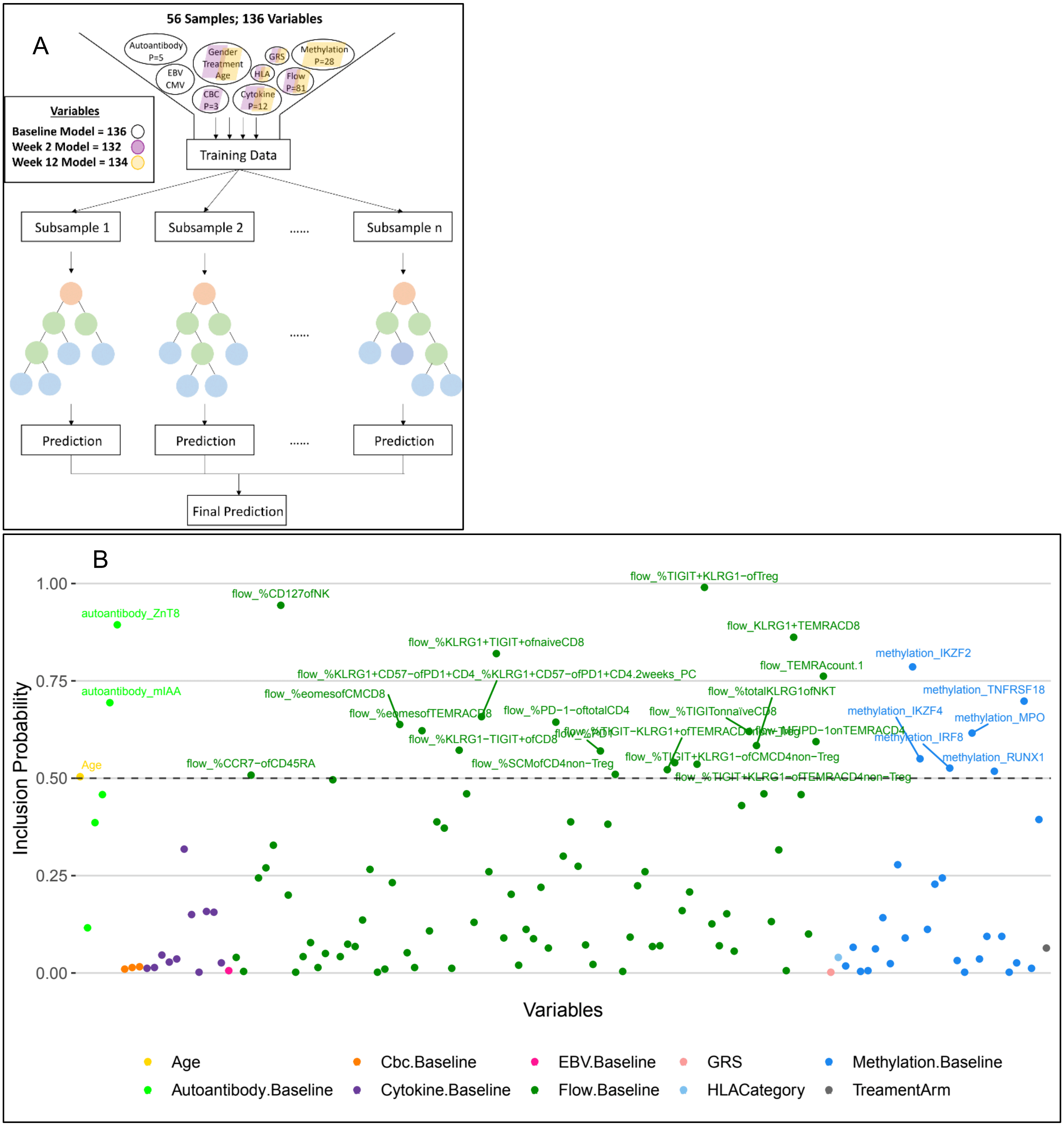
A
56 Samples; 136 Variables
Autoantibody P=5
EBV CMV
Gender Treatment Age
GRS
HLA
Methylation P=28
Flow P=81
CBC P=3
Cytokine P=12
Variables
Baseline Model = 136
Week 2 Model = 132
Week 12 Model = 134
Training Data
Subsample 1
Subsample 2
Subsample n
Prediction
Final Prediction
B
Inclusion Probability
Variables
1.00
0.75
0.50
0.25
0.00
flow_%TIGIT+KLRG1−ofTreg
flow_%CD127ofNK
autoantibody_ZnT8
flow_KLRG1+TEMRACD8
flow_%KLRG1+TIGIT+ofnaiveCD8
methylation_IKZF2
flow_TEMRAcount.1
flow_%KLRG1+CD57−ofPD1+CD4_%KLRG1+CD57−ofPD1+CD4.2weeks_PC
autoantibody_mIAA
flow_%eomesofCMCD8
flow_%totalKLRG1ofNKT
methylation_TNFRSF18
flow_%eomesofTEMRACD8
flow_%PD−1−oftotalCD4
flow_%TIGITonnaïveCD8
methylation_IKZF4
methylation_MPO
flow_%KLRG1−TIGIT+ofCD8
methylation_IRF8
Age
flow_%CCR7−ofCD45RA
flow_%SCMofCD4non−Treg
flow_%TIGIT+KLRG1−ofCMCD4non−Treg
methylation_RUNX1
Age
Autoantibody.Baseline
Cbc.Baseline
Cytokine.Baseline
EBV.Baseline
Flow.Baseline
GRS
HLACategory
Methylation.Baseline
TreamentArm

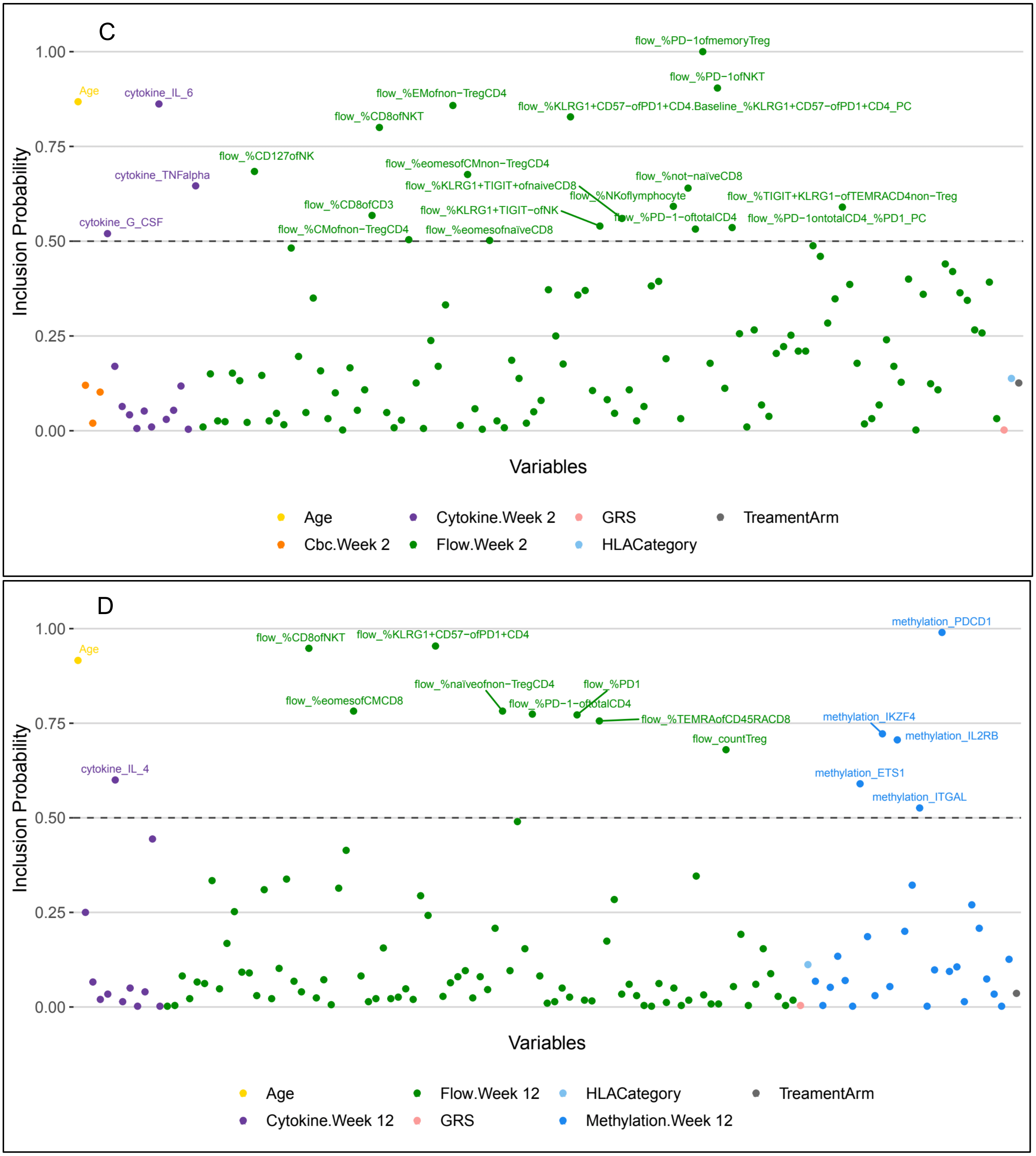
C
1.00
0.75
0.50
0.25
0.00
Inclusion Probability
Age
cytokine_IL_6
flow_%PD−1ofmemoryTreg
flow_%PD−1ofNKT
flow_%EMofnon−TregCD4
flow_%KLRG1+CD57−ofPD1+CD4.Baseline_%KLRG1+CD57−ofPD1+CD4_PC
flow_%CD8ofNKT
flow_%CD127ofNK
cytokine_TNFalpha
flow_%eomesofCMnon−TregCD4
flow_%not−naïveCD8
flow_%KLRG1+TIGIT+ofnaiveCD8
flow_%NKoflymphocyte
flow_%TIGIT+KLRG1−ofTEMRACD4non−Treg
flow_%CD8ofCD3
flow_%KLRG1+TIGIT−ofNK
flow_%PD−1−oftotalCD4
flow_%PD−1ontotalCD4_%PD1_PC
cytokine_G_CSF
flow_%CMofnon−TregCD4
flow_%eomesofnaïveCD8
Variables
Age
Cytokine.Week 2
GRS
TreamentArm
Cbc.Week 2
Flow.Week 2
HLACategory
D
1.00
0.75
0.50
0.25
0.00
Inclusion Probability
Age
flow_%CD8ofNKT
flow_%KLRG1+CD57−ofPD1+CD4
methylation_PDCD1
flow_%naïveofnon−TregCD4
flow_%PD1
flow_%eomesofCMCD8
flow_%PD−1−oftotalCD4
flow_%TEMRAofCD45RACD8
methylation_IKZF4
methylation_IL2RB
flow_countTreg
cytokine_IL_4
methylation_ETS1
methylation_ITGAL
Variables
Age
Flow.Week 12
HLACategory
TreamentArm
Cytokine.Week 12
GRS
Methylation.Week 12

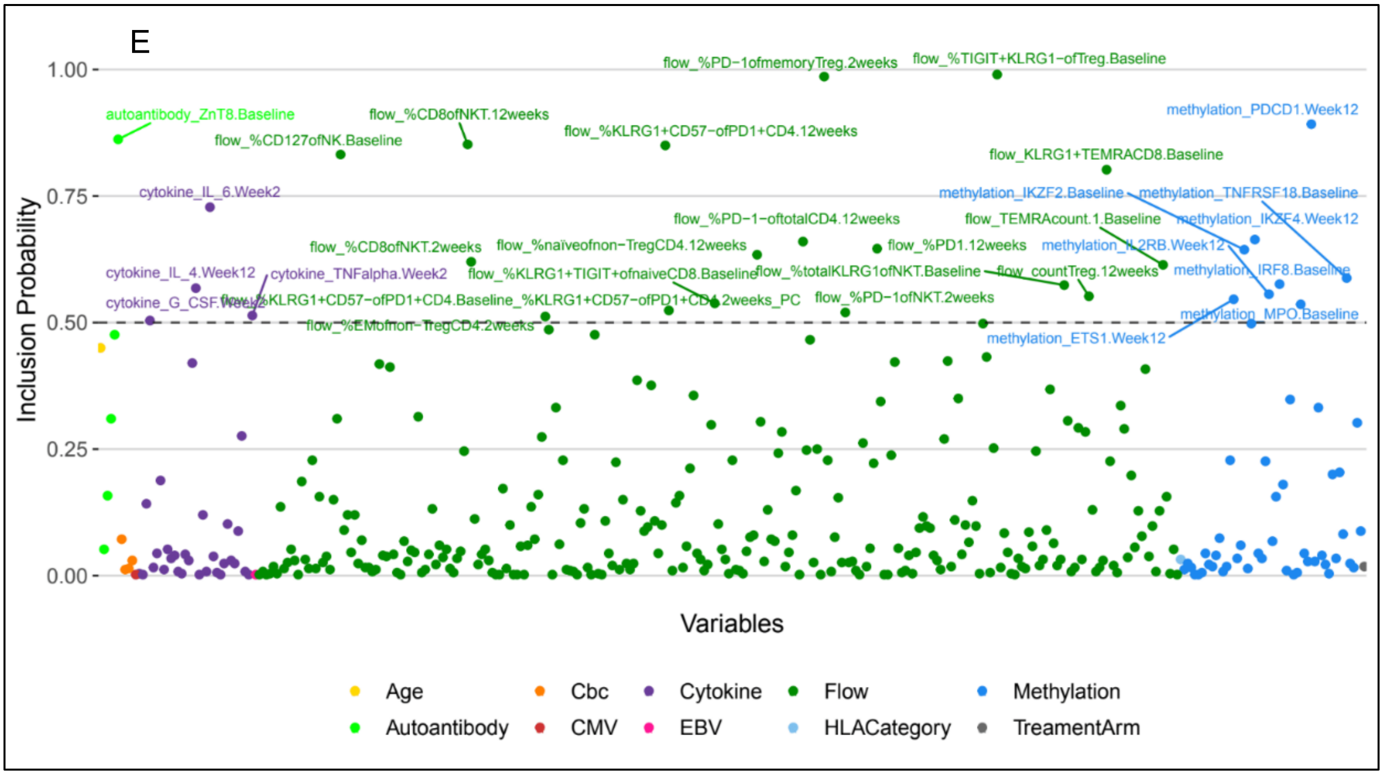

<u>Supplemental Figure 7: Random Forest model exploring a responder signature to ATG and ATG/G-CSF from available mechanistic assays.</u> (A) Depiction of clinical and immunologic variables into training data sets, undergoing random forest modeling *n*th times to produce 500 prediction models for inclusion probability to identify ATG and ATG/G-CSF responders using data from (B) baseline time point only, (C) 2 weeks only, (D) 12 weeks only, and (E) all three timepoints combined. If two variables are intercorrelated then the principal component of those two variables was used.

Supplemental Table 1: **Differential expression via bulk RNA-seq.** Comparisons were made between each treatment arm versus placebo as well as between ATG responders/non-responders and ATG/G-CSF responders/non-responders at 2 and 12 weeks. Benjamini-Hochberg-adjusted p-value (adjusted p); p<0.05*, p<0.001**.

A

ATG vs Placebo (Ref.) at Week 2

Gene	baseMean	Log2FC	p	Adjusted p	
CCR4	17.05	−1.71	<0.001	<0.001	**
CD40LG	44.38	−1.80	<0.001	<0.001	**
CTLA4	13.35	−0.30	0.313	0.398	
CXCL10	13.22	−1.31	0.044	0.088	
CXCR3	156.99	0.04	0.801	0.862	
EOMES	81.32	−0.32	0.082	0.144	
FOXP3	16.10	−0.33	0.236	0.331	
GATA3	75.38	−0.58	0.001	0.003	*
IKZF2	51.24	−0.31	0.095	0.148	
IL2RA	20.16	−0.98	<0.001	<0.001	**
ITGAL	648.51	−0.04	0.57	0.665	
MPO	155.47	3.20	<0.001	<0.001	**
TBX21	114.58	−0.47	0.011	0.025	*
TNFRSF18	27.76	0.04	0.869	0.869	

B

ATG vs Placebo (Ref.) at Week 12

Gene	baseMean	Log2FC	p	Adjusted p	
CCR4	18.75	−0.03	0.921	0.960	
CD40LG	58.58	−0.59	0.001	0.009	*
CTLA4	12.54	0.02	0.960	0.960	
CXCL10	15.04	0.06	0.866	0.960	
CXCR3	178.37	0.12	0.274	0.547	
EOMES	105.01	0.34	0.014	0.048	*
FOXP3	21.38	−0.56	0.010	0.048	*
GATA3	91.12	−0.17	0.086	0.241	
IKZF2	66.85	0.08	0.537	0.873	
IL2RA	27.06	−0.21	0.272	0.547	
ITGAL	737.44	0.13	0.012	0.048	*
MPO	53.78	0.04	0.915	0.960	
TBX21	150.46	0.01	0.914	0.960	
TNFRSF18	32.58	−0.12	0.561	0.873	

C

ATG/GCSF vs Placebo (Ref.) at Week 2

Gene	baseMean	Log2FC	p	Adjusted p	
CCR4	17.05	−1.42	<0.001	<0.001	**
CD40LG	44.38	−1.92	<0.001	<0.001	**
CTLA4	13.35	−0.53	0.079	0.111	
CXCL10	13.22	−0.42	0.525	0.612	
CXCR3	156.99	−0.08	0.621	0.669	
EOMES	81.32	−0.50	0.008	0.014	*
FOXP3	16.10	−0.63	0.028	0.043	*
GATA3	75.38	−0.62	<0.001	0.001	*
IKZF2	51.24	−0.53	0.005	0.01	*
IL2RA	20.16	−0.99	<0.001	<0.001	**
ITGAL	648.51	−0.07	0.278	0.353	
MPO	155.47	2.30	<0.001	<0.001	**
TBX21	114.58	−0.78	<0.001	<0.001	**
TNFRSF18	27.76	−0.03	0.89	0.89	

D

ATG/GCSF vs Placebo (Ref.) at Week 12

Gene	baseMean	Log2FC	p	Adjusted p	
CCR4	18.75	−0.32	0.37	0.471	
CD40LG	58.58	−0.92	<0.001	<0.001	**
CTLA4	12.54	−0.10	0.762	0.762	
CXCL10	15.04	0.57	0.151	0.264	
CXCR3	178.37	−0.14	0.248	0.385	
EOMES	105.01	0.22	0.12	0.241	
FOXP3	21.38	−0.64	0.004	0.014	*
GATA3	91.12	−0.43	<0.001	<0.001	**
IKZF2	66.85	−0.09	0.515	0.601	
IL2RA	27.06	−0.32	0.107	0.241	
ITGAL	737.44	0.10	0.061	0.171	
MPO	53.78	1.18	0.001	0.002	*
TBX21	150.46	−0.13	0.35	0.471	
TNFRSF18	32.58	0.11	0.606	0.653	

E

ATG: Responder vs Non−Responder (Ref.) at Week 2

Gene	baseMean	Log2FC	p	Adjusted p
CCR4	17.05	1.89	0.004	0.052
CD40LG	44.38	0.43	0.267	0.535
CTLA4	13.35	0.39	0.461	0.687
CXCL10	13.22	0.80	0.539	0.687
CXCR3	156.99	0.17	0.497	0.687
EOMES	81.32	0.52	0.107	0.457
FOXP3	16.10	−0.08	0.868	0.868
GATA3	75.38	0.35	0.246	0.535
IKZF2	51.24	0.28	0.401	0.687
IL2RA	20.16	−0.20	0.605	0.706
ITGAL	648.51	0.17	0.145	0.457
MPO	155.47	0.14	0.833	0.868
TBX21	114.58	0.45	0.163	0.457
TNFRSF18	27.76	0.69	0.087	0.457

F

ATG: Responder vs Non−Responder (Ref.) at Week 12

Gene	baseMean	Log2FC	p	Adjusted p
CCR4	18.75	0.67	0.234	0.829
CD40LG	58.58	0.08	0.785	0.873
CTLA4	12.54	0.08	0.873	0.873
CXCL10	15.04	−0.21	0.741	0.873
CXCR3	178.37	−0.04	0.814	0.873
EOMES	105.01	0.14	0.545	0.873
FOXP3	21.38	−0.34	0.343	0.873
GATA3	91.12	−0.11	0.527	0.873
IKZF2	66.85	−0.19	0.374	0.873
IL2RA	27.06	0.46	0.146	0.829
ITGAL	737.44	0.15	0.066	0.829
MPO	53.78	0.29	0.591	0.873
TBX21	150.46	0.04	0.845	0.873
TNFRSF18	32.58	0.39	0.237	0.829

G

ATG/GCSF: Responder vs Non-Responder (Ref.) at Week 2

Gene	baseMean	Log2FC	p	Adjusted p
CCR4	17.05	−0.55	0.310	0.676
CD40LG	44.38	−0.73	0.036	0.247
CTLA4	13.35	0.24	0.611	0.778
CXCL10	13.22	1.03	0.368	0.676
CXCR3	156.99	−0.44	0.058	0.247
EOMES	81.32	−0.22	0.435	0.676
FOXP3	16.10	−0.39	0.386	0.676
GATA3	75.38	−0.51	0.060	0.247
IKZF2	51.24	−0.25	0.401	0.676
IL2RA	20.16	−0.04	0.921	0.940
ITGAL	648.51	0.19	0.071	0.247
MPO	155.47	0.14	0.811	0.940
TBX21	114.58	0.02	0.940	0.940
TNFRSF18	27.76	−0.18	0.607	0.778

H

ATG/GCSF: Responder vs Non-Responder (Ref.) at Week 12

Gene	baseMean	Log2FC	p	Adjusted p
CCR4	18.75	−0.30	0.582	0.903
CD40LG	58.58	0.26	0.343	0.903
CTLA4	12.54	−0.62	0.229	0.903
CXCL10	15.04	0.91	0.131	0.903
CXCR3	178.37	−0.08	0.645	0.903
EOMES	105.01	−0.02	0.916	0.916
FOXP3	21.38	0.47	0.175	0.903
GATA3	91.12	−0.10	0.541	0.903
IKZF2	66.85	−0.05	0.823	0.916
IL2RA	27.06	0.10	0.744	0.916
ITGAL	737.44	−0.05	0.514	0.903
MPO	53.78	−0.43	0.400	0.903
TBX21	150.46	0.03	0.880	0.916
TNFRSF18	32.58	−0.18	0.562	0.903

Supplemental Table 2: Flow cytometry panels antibodies and cell type markers.

**Marker-Dye**	**Clone**	**Fluorochrome**	**Company**	**Laser-Detector**	**Catalog**	**Research Resource Identifiers (RRIDs)**
Live/Dead Blue		BUV	Invitrogen/Thermo Fisher	355-450/50	L23105	N/A
**Panel 1**						
CD56	NCAM16.2	BUV395	BD Biosciences	355-379/28	563554	AB_2687886
CD45RA	HI100	BUV737	BD Biosciences	355-740/35	612846	AB_2870168
Ki67	Ki-67	BV421	BioLegend	405-450/50	350506	AB_2563860
CCR7	G043H7	BV510	BioLegend	405-525/50	353232	AB_2563866
CD3	OKT3	evolve605	eBioscience/Invitrogen	405-605/12	N/A	N/A
PD1	EH12.2H7	BV650	BioLegend	405-655/8	329950	AB_2566362
CD127	A019D5	BV711	BioLegend	405-710/50	351328	AB_2562908
CD45R0	UCHL1	BV786	BD Biosciences	405-780/60	564290	AB_2738733
CD4	RPA-T4	BB515	BD Biosciences	488-530/30	564419	AB_2744419
Eomes	WD1928	PE	Invitrogen/Thermo Fisher	561-582/15	12-4877-42	AB_2572615
FoxP3	259D/C7	PE-CF594	BD Biosciences	561-610/20	562421	AB_11153143
KLRG1	REA261	PE-vio770	Miltenyi	561-780/60	N/A	N/A
TIGIT	MBSA43	APC	Invitrogen / Thermo Fisher	640-660/20	17-9500-42	AB_2573305
CD8	SK1	AF700	BioLegend	640-710/50	344724	AB_2562790
CD57	REA769	APC-vio770	Miltenyi	640-780/60	130-111-813	AB_2658756
**Panel 2**						
CD8	SK1	BUV737	BD Biosciences	355-740/35	612755	AB_2870086
CD4	SK3	eVolve655	eBioscience/Invitrogen	405-655/8	N/A	N/A
CD57	REA769	APC-Vio770	Miltenyi	640-780/60	130-111-813	AB_2658756
CD38	HB-7	PE-Cy7	BioLegend	561-780/60	356608	AB_2561904
CCR6	G034E3	BV510	BioLegend	405-525/50	353424	AB_2563868
CD45RA	HI100	BV605	BioLegend	405-605/12	304134	AB_2563814
CXCR5	J252D4	AF647	BioLegend	640-660/20	356906	AB_2561815
CD3	HIT3a	AF700	BioLegend	640-710/50	300324	AB_493739
CCR7	3D12	BUV395	BD Biosciences	355-379/28	740267	AB_2740009

PD-1	EH12.2H7	BV421	BioLegend	405-450/50	329920	AB_10960742
CD95	DX2	FITC	BioLegend	488-530/30	305606	AB_314544
CD27	O323	BV711	BioLegend	405-710/50	302834	AB_2563809
CD45RO	UCHL1	PerCP-Cy5.5	BioLegend	488-710/50	304222	AB_2174124
CD127	A019D5	BV785	BioLegend	405-780/60	351330	AB_2563605
CXCR3	1C6/CXCR3	PE-Cy5	BD Biosciences	561-670/30	551128	AB_394061
ICOS	C398.4A	PE	BioLegend	561-582/15	313508	AB_416332
FOXP3	259D/C7	PE-CF594	BD Biosciences	561-610/20	562421	AB_11153143

**Cell Subsets**	**Marker designation**
CD4 T cell	CD3^{+} CD56^{-} CD4^{+}
Exhausted CD4 T cells	PD1^{+} KLRG1^{+} CD57^{-} CD4^{+}
Senescent CD4 T cells	CD57^{+}KLRG1^{+} of PD-1^{-} CD4^{+}
Conventional T cell / Teff / CD4 Non-Treg	CD4^{+} CD127^{high} FOXP3^{-}
Treg	CD4^{+} CD127$^{lo/-}$ FOXP3^{+}
Memory Treg	CD45RO^{+} CD4^{+} CD127$^{lo/-}$ FOXP3^{+}
CD8 T cell	CD3^{+} CD56^{-} CD8^{+}
Naïve CD8 T cell	CD45RA^{+} CD8^{+}
Hyporesponsive / Senescent CD8 T cells	PD1^{-} KLRG1^{+} CD57^{-} CD8^{+}
TEMRA CD8	CD8^{+} CD45RA^{+} CCR7^{-}
